# Uncovering the secretome of mesenchymal stromal cells exposed to healthy, traumatic, and degenerative intervertebral discs: a proteomic analysis

**DOI:** 10.1186/s13287-020-02062-2

**Published:** 2021-01-07

**Authors:** Sebastian Wangler, Amir Kamali, Christina Wapp, Karin Wuertz-Kozak, Sonja Häckel, Claudia Fortes, Lorin M. Benneker, Lisbet Haglund, R. Geoff Richards, Mauro Alini, Marianna Peroglio, Sibylle Grad

**Affiliations:** 1 AO Research Institute Davos, Clavadelerstrasse 8, 7270 Davos, Switzerland; 2 Department of Orthopaedic Surgery and Traumatology, Inselspital, Bern University Hospital, University of Bern, Bern, Switzerland; 3 Department of Health Sciences and Technology, ETH Zurich, Zurich, Switzerland; 4 Department of Biomedical Engineering, Rochester Institute of Technology (RIT), Rochester, NY USA; 5 Schön Clinic Munich Harlaching, Spine Center, Academic Teaching Hospital and Spine Research Institute of the Paracelsus Medical University Salzburg (Austria), Munich, Germany; 6 Functional Genomics Center Zurich, Zurich, Switzerland; 7 Department of Surgery, Division of Orthopaedics, Faculty of Medicine, McGill University, Montreal, Canada

**Keywords:** Mesenchymal stromal cells, Secretome, Intervertebral disc, Conditioned medium, Intervertebral disc degeneration

## Abstract

**Background:**

Mesenchymal stromal cells (MSCs) have been introduced as promising cell source for regenerative medicine. Besides their multilineage differentiation capacity, MSCs release a wide spectrum of bioactive factors. This secretome holds immunomodulatory and regenerative capacities. In intervertebral disc (IVD) cells, application of MSC secretome has been shown to decrease the apoptosis rate, induce proliferation, and promote production of extracellular matrix (ECM). For clinical translation of secretome-based treatment, characterization of the secretome composition is needed to better understand the induced biological processes and identify potentially effective secretomes.

**Methods:**

This study aimed to investigate the proteome released by bone marrow-derived MSCs following exposure to a healthy, traumatic, or degenerative human IVD environment by mass spectroscopy and quantitative immunoassay analyses. Exposure of MSCs to the proinflammatory stimulus interleukin 1β (IL-1β) was used as control.

**Results:**

Compared to MSC baseline secretome, there were 224 significantly up- or downregulated proteins following healthy, 179 following traumatic, 223 following degenerative IVD, and 160 proteins following IL-1β stimulus. Stimulation of MSCs with IVD conditioned media induced a more complex MSC secretome, involving more biological processes, compared to stimulation with IL-1β. The MSC response to stimulation with IVD conditioned medium was dependent on their pathological status.

**Conclusions:**

The MSC secretome seemed to match the primary need of the IVD: homeostasis maintenance in the case of healthy IVDs, versus immunomodulation, adjustment of ECM synthesis and degradation disbalance, and ECM (re) organization in the case of traumatic and degenerative IVDs. These findings highlight the importance of cell preconditioning in the development of tailored secretome therapies.

**Graphical abstract:**

The secretome of human bone marrow-derived mesenchymal stromal cells (MSCs) stimulated with intervertebral disc (IVD) conditioned medium was analyzed by proteomic profiling. Depending on the pathological state of the IVD, the MSC secretome protein composition indicated immunomodulatory or anabolic activity of the secretome. These findings may have implications for tailored secretome therapy for the IVD and other tissues. 
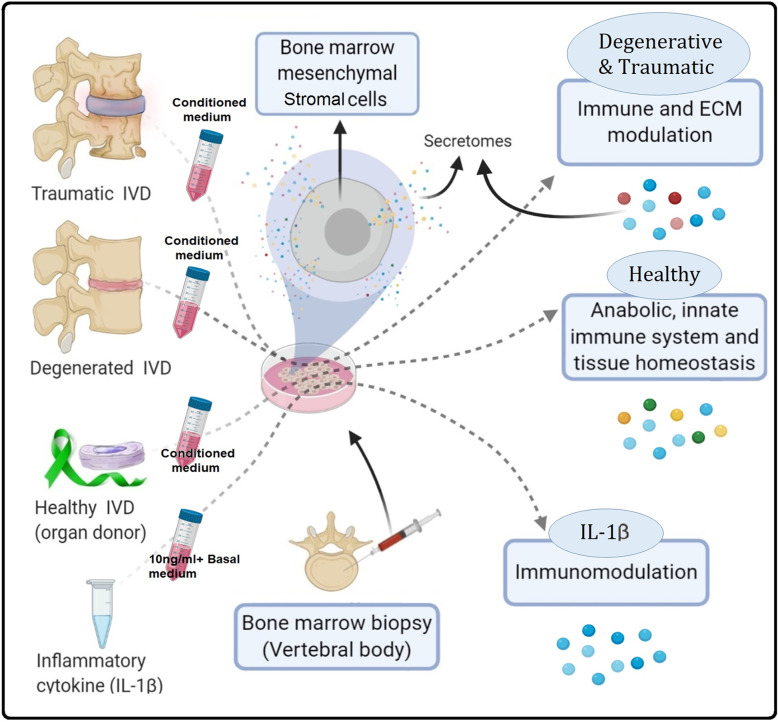

## Background

Mesenchymal stromal cells (MSCs) have been the subject of extended research in the field of regenerative medicine. Defined as fibroblast-like non-hematopoietic cells by the International Society for Stem Cell Research (ISSCR), this pluripotent cell population holds differentiation potential towards various lineages [1]. In the field of musculoskeletal regeneration, MSCs have gained interest for their ability to differentiate towards chondrogenic and osteogenic phenotypes [2]. In the intervertebral disc (IVD), injection of naïve MSCs into degenerated IVDs has been associated with a regenerative effect, both in preclinical and clinical trials [3, 4]. However, the exact underlying mechanism remains unknown. On the one hand, host IVD cells can induce a discogenic phenotype in implanted MSCs, as evidenced by upregulated gene expression and synthesis of extracellular matrix (ECM) molecules. On the other hand, MSCs can influence IVD cells by secreting bioactive factors which cause a shift from a degenerative towards a healthier disc cell phenotype [5–7]. Nonetheless, in a degenerative IVD, MSCs face a challenging hypoxic and acidic milieu with limited nutrient supply [8, 9]. In vivo studies indicated that MSC survival under such conditions is limited [10, 11]. It is therefore hypothesized that the main regenerative effect of MSCs would be mediated by paracrine stimulation rather than by engrafting, differentiation, and de novo ECM production [12].

Paracrine stimulation is mediated by substances secreted by the MSCs as a response to the perceived environment. The secreted bioactive substances can be termed secretome. Released factors include soluble proteins, free nucleic acids, lipids, and extracellular vesicles which can be further subdivided into apoptotic bodies, micro-particles, and exosomes [13]. The secretome from MSCs is believed to hold great potential for tissue repair and regenerative medicine [14–17]. Application of naïve MSC secretome in animal models has shown to significantly improve the pathology of various diseases such as graft versus host disease, autoimmune, and inflammatory diseases [18]. Clinical application of naïve MSC secretome has already been investigated in small patient groups suffering from alveolar bone atrophy, alopecia, or skin damage following ablative fractional carbon dioxide laser resurfacing; in all patient groups, application of MSC secretome led to improved recovery, with no reported adverse effects [19–21].

The secretome composition is influenced by the environment which the MSCs are exposed to [17, 18, 22]. For instance, hypoxic preconditioning was associated with increased production of growth factors, including vascular endothelial growth factor (VEGF), fibroblast growth factor 2 (FGF-2), hepatocyte growth factor (HGF), and insulin-like growth factor 1 (IGF-1) [23]. Exposure to an inflammatory stimulus such as interleukin 1-beta (IL-1β), tumor necrosis factor-alpha (TNF-α), or interferon-gamma (IFN-γ) was shown to initiate the production of immune-modulatory factors. These include granulocyte colony-stimulating factor (G-CSF) [24], factor H which inhibits complement activation [25], and galectin-9 which suppresses T-cell proliferation [26], among others. Interestingly, culturing of MSCs in three-dimensional (3D) arrangement was also associated with an induced secretion of different potentially therapeutic factors compared to two-dimensional (2D) culture, including G-CSF, VEGF, IL-1 receptor antagonist (IL-1Ra), or FGF-1 [27–29]. Preconditioning of adipose-derived stem cells (ASC) with lipopolysaccharide resulted in the production of a secretome that was superior in hepatic regeneration compared to the secretome of unstimulated ASC [30]. Also, preconditioning of ASC with TNF-α potentiated the exosome efficacy for bone regeneration [31]. Furthermore, in vivo mouse models indicated accelerated skin wound healing following application of secretome from MSCs primed by hypoxia compared to MSC secretome obtained under normoxic conditions [32].

Concerning the IVD, so far only the application of unprimed MSC secretome (mainly in form of extracellular vesicles) has been investigated as a potential cell-free treatment strategy. The studies described the beneficial effect of secretome application on IVD cells, including prevention of cell death, decrease in apoptosis rate, enhanced cell proliferation, and ECM production [33]. The composition of the secretome, however, remains unknown. Aiming for clinical translation of secretome-based treatments, characterization of the secretome composition is needed to better understand its biological effect.

**Fig. 1:**
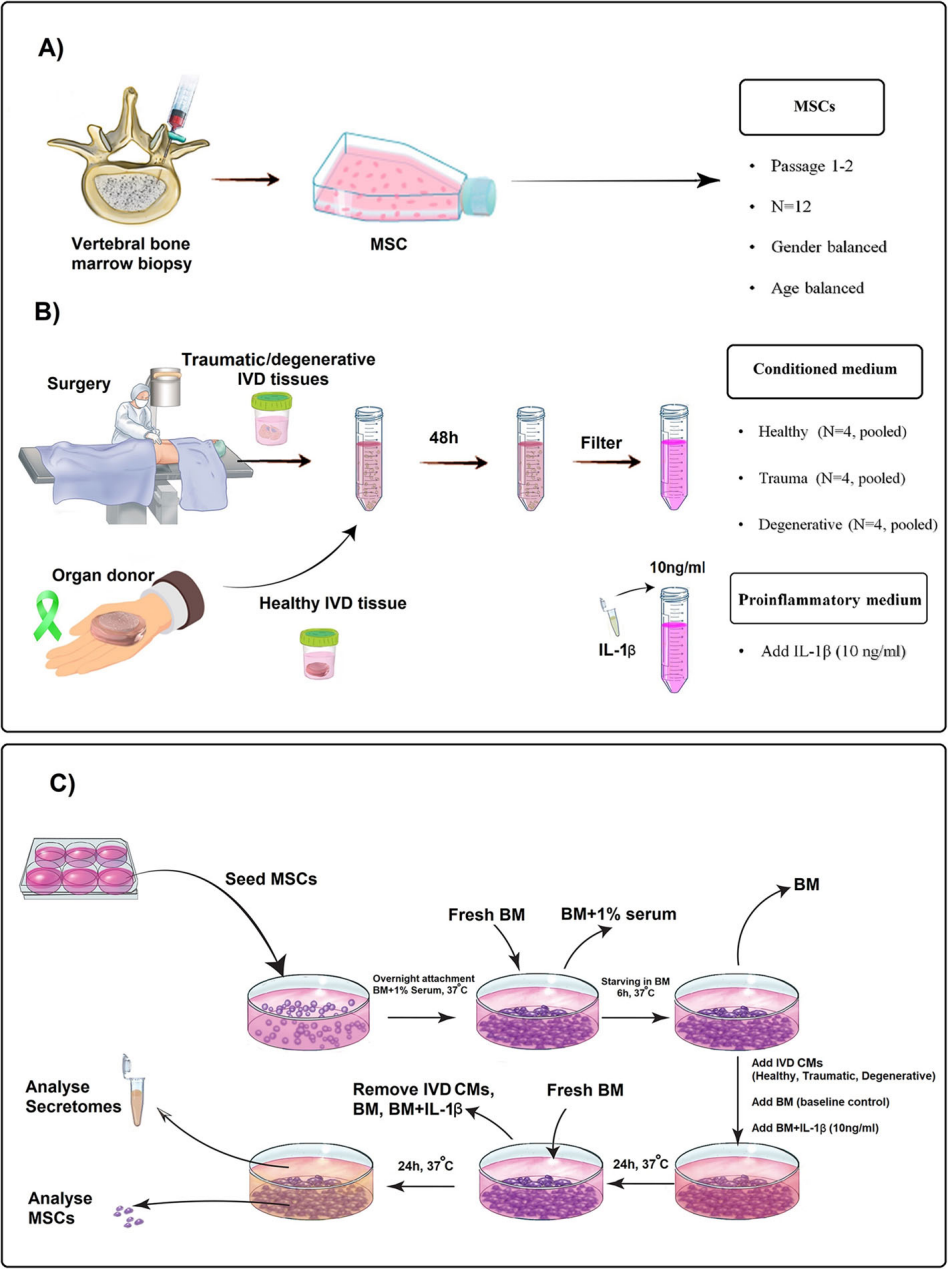
Experimental setup. **a** Mesenchymal stromal cells (MSCs; *N* = 12) were isolated from vertebral bone marrow aspirates obtained with written consent from patients undergoing spine surgery. **b** Intervertebral disc (IVD) tissue from patients suffering from spinal trauma (referred to as traumatic), from patients with disc degeneration (referred to as degenerative), and non-degenerated IVDs from organ donors (referred to as healthy) were obtained with written patient and/or familial consent. Tissue was incubated in basal medium for 48 h to collect released factors (referred to as IVD conditioned medium (CM)). Basal medium supplemented with IL-1β (10 ng/mL) was prepared as proinflammatory control. **c** MSCs were seeded in 6-well plates. After overnight attachment and 6 h of starvation, MSCs were stimulated with healthy CM (*N* = 4, pooled), traumatic CM (*N* = 4, pooled), degenerative CM (*N* = 4, pooled), IL-1β, and basal medium (baseline control), respectively. After 24 h of stimulation, stimulants were removed, and fresh basal medium was added to collect the MSC secretome during the following 24 h. MSC secretome was analyzed by LC-MS/MS and immunoassay. MSCs were analyzed by CellTiter-Blue, lactate dehydrogenase (LDH) assay, DNA quantification. BM = basal medium (low glucose-DMEM, 1% L-Ascorbic acid 2-phosphate, 1% Glutamax)

In the present study, we analyzed the protein composition within the secretome of MSCs exposed to healthy, traumatic, and degenerative human IVD conditioned medium. Exposure to a supraphysiological concentration of IL-1β was further used as a pro-inflammatory priming control. We hypothesized that distinct differences existed between the protein profiles of secretomes from MSCs primed with different IVD conditioned media or IL-1β as a single pro-inflammatory stimulus. Proteomic profiling by mass spectroscopy (LC-MS/MS) and quantitative immunoassays were used to identify proteins within the MSC secretome. Gene set enrichment analysis (GSEA) allowed us to identify enriched biological processes in MSCs following the different priming strategies (Fig. 1).

## Methods

### MSC isolation and expansion

MSCs were isolated by Ficoll® gradient centrifugation and adherence to tissue culture plastic from vertebral bone marrow aspirates obtained with written consent from patients undergoing spine surgery. Standardized methods were applied for cell isolation as previously described [34, 35]. MSCs from 12 different donors were used for this study (Suppl. Fig. 1A). Cells were expanded in growth medium composed of alpha minimal essential medium (α-MEM, Gibco) supplemented with 10% fetal bovine serum (FBS+, Sera Plus, Pan Biotech), 1% penicillin-streptomycin (P/S, 100x, Gibco), and 5 ng/mL FGF-2 (Fitzgerald Industries) according to standardized procedures [36, 37]. Passage 3 MSCs were used in this study.

### IVD conditioned medium

Human IVD tissues from patients with traumatic injury (“traumatic” sample) and from patients diagnosed with IVD degeneration (“degenerative” sample) were obtained with written consent from patients undergoing spine surgery. Non-degenerated (“healthy” sample) IVD tissues were obtained from organ donors after donor and familial consent by the McGill Scoliosis & Spinal Research Group via a collaboration with Transplant Quebec and approval by the McGill University’s Institutional Review Board (IRB# A04-M53-08B). Human IVDs from organ donors, degenerative and traumatic patients were used to produce IVD conditioned medium (CM) as previously described (Suppl. Fig. 1B) [38]. Briefly, the tissue was weighed and washed in red cell lysis buffer for 5 min. Tissue was then washed three times in phosphate buffered saline (PBS) supplemented with 1% P/S. Cartilaginous endplates were removed and IVD tissue was cut into pieces (approximately 4 × 4 × 4 mm). Basal medium, low glucose (1 g/L) Dulbecco’s modified Eagle’s medium (lg-DMEM, Gibco) supplemented with L-ascorbic acid 2-phosphate sesquimagnesium salt hydrate (50 μg/mL, Sigma-Aldrich), 1% Glutamax (Gibco), and 0.1% Primocin™ (InvivoGen), was added to the tissue (3.5 mL/g tissue) in a 50-mL centrifugation tube and incubated for 48 h. Then, CM was filtered through a 100 μm pore size cell strainer (Falcon™) and aliquoted into 2 mL low binding protein tubes. Aliquots were stored at − 80 °C until use.

### MSC stimulation and secretome collection

MSCs were plated in 6-well plates at a density of 10,000/cm^2^ and cultured in growth medium for 14 h. Following cell attachment, cells were washed 3 times with 1 mL PBS and subsequently starved for 6 h in 1 mL basal medium. Basal medium was removed and 1 mL of pooled IVD CMs (*N* = 4 for each degenerative, traumatic, or healthy CM) was added for MSC stimulation. As a pro-inflammatory positive control, cells were stimulated with 1 mL of basal medium containing 10 ng/mL IL-1β (PeproTech). As baseline control, cells were incubated with 1 mL of basal medium only. After 24 h, medium was removed, and cells were washed 3 times with 1 mL of lg-DMEM. To generate the secretome, 1 mL of basal medium without Primocin™ was added to each well. After 24 h, MSC secretome was collected in low binding protein tubes and stored at − 80 °C; MSCs were analyzed microscopically, for metabolic activity, lactate dehydrogenase (LDH) activity, and DNA content.

### Metabolic activity

The resazurin-based CellTiter-Blue™ Cell Viability Assay (Promega) was used to measure the MSCs metabolic activity. Prior to the assay, MSCs were washed with 1 mL PBS. Subsequently, 1 mL of basal medium (without Primocin™) and 200 μL of CellTiter-Blue™ were added. Fluorescence intensity was measured after 3 h incubation using a VICTOR™ multilabel plate reader (Perkin Elmer). Values were normalized to the baseline treatment condition for each MSC donor.

### Lactate dehydrogenase measurement

MSC viability was assessed using the LDH based cytotoxicity detection KitPlus™ (Roche) after secretome collection according to the manufacturer’s instructions. As a cytotoxic positive control, cells were treated with 1% Triton X-100 (Sigma-Aldrich) in basal medium without Primocin™. For the negative control, cells were left untreated with basal medium without Primocin™. Absorbance was measured at 490 nm using the VICTOR™ multilabel plate reader. For each MSC donor, cytotoxicity was calculated by dividing the difference of the sample and the negative control by the difference of the positive control and the negative control. This resulted in the negative control having 0% and the positive control 100% cytotoxicity.

### DNA quantification

MSCs were digested with 500 μL of proteinase K (0.5 mg/mL, Roche) at 56 °C for 16 h. DNA quantification was performed with Qubit® 4 Fluorometer (Invitrogen) using the QubitR dsDNA HS assay kit according to the manufacturer’s instructions. DNA content after secretome collection was normalized to the DNA amount of the attached cells 14 h after seeding.

### Cell morphology

For each condition and donor, microscopic images of one well of the 6-well plate were taken immediately before secretome collection using a × 5 magnification (Axiovert 40 CFL, Zeiss).

### Sample processing for LC-MS/MS analysis

MSC secretomes from all 12 donors for all treatment conditions (healthy, degenerative, traumatic, baseline, and IL-1β) were analyzed. The samples were collected and measured in two batches of 48 samples (traumatic, degenerative, IL-1β, baseline) and 24 samples (healthy and baseline). In both batches, baseline samples were included to account for differences between batches. For each sample, the protein concentration was measured using the Qubit® Protein Assay Kit (Life Technologies, Switzerland). The samples were then prepared by using a commercial iST Phonix Kit (PreOmics, Germany). Mass spectrometry analysis was performed on a Q-Exactive HF-X mass spectrometer (Thermo Scientific) equipped with a Digital PicoView source (New Objective) and coupled to an M-Class UPLC (Waters). Solvent composition at the two channels was 0.1% formic acid for channel A and 0.1% formic acid, 99.9% acetonitrile for channel B. For each sample, 4 μL of peptides were loaded on a commercial MZ Symmetry C18 Trap Column (100 Å, 5 μm, 180 μm × 20 mm, Waters) followed by nanoEase MZ C18 HSS T3 Column (100 Å, 1.8 μm, 75 μm × 250 mm, Waters). The peptides were eluted at a flow rate of 300 nL/min by a gradient from 8 to 27% B in 82 min, 35% B in 5 min, and 80% B in 1 min. Samples were acquired in a randomized order. Only precursors with intensity above 110,000 were selected for MS/MS. 

### Quantification of chemokines and cytokines

As a complementary analysis technique, multiplex immunoassay was used to quantify cytokines and chemokines present in pooled IVD CM and released by MSCs following stimulation by IVD CM. All samples were analyzed with the proinflammatory panel 1, cytokine panel 1, and chemokine panel 1 assay kits (Meso Scale Discovery (MSD), Rockville, MD, USA) following the manufacturer’s protocol. Briefly, standards and samples (50 μL) were added to each well in technical duplicates. Plates were sealed and incubated under shaking for 2 h at room temperature (RT). Then, plates were washed, 25 μL of detection antibody solution was added to each well, and plates were incubated under shaking for 2 h at RT. After the final washing, 150 μL of MSD read buffer T (× 2) was added to each well and the plates were read using the MESO QuickPlex SQ 120 instrument (MSD). The concentration of each analyte in MSC secretomes was normalized to the DNA content of the respective sample. Finally, all values were normalized to the baseline control sample for each donor, and fold changes were calculated.

### Bioinformatics and statistical analyses

We performed MS1 intensity-based label-free quantification to estimate protein fold changes among conditions. The acquired raw MS data were processed by MaxQuant (version 1.6.2.3), followed by protein identification using the integrated Andromeda search engine. Spectra were searched against a Uniprot human reference proteome (taxonomy 9906, canonical version from 2016-12-09), concatenated to its reversed decoyed fasta database and common protein contaminants. A peptide was considered quantified if it was observed in more than 50% of samples within one of the conditions. In addition, two quantified peptides per protein were required; proteins with only one quantified peptide were excluded.

Normalization was applied to remove systematic differences in protein abundance due to different amounts of sample loaded on the column. The *z*-score of the log2 transformed intensities was computed and updated by the average standard deviation of the log2 transformed intensities in all samples to preserve the original scale of the measurement. After normalization, all samples showed a similar distribution. As a next step, to obtain protein fold change among conditions, peptide intensities were modeled using a mixed linear model [39], to account for the repeated measures design. Log2 fold changes were computed based on the model parameters using the R package lmerTest [39]. The baseline samples were used as reference condition in all comparisons.

For the analysis R version 3.5.1 (2018-07-02) with the platform x86_64-w64-mingw32/x64 (64 bit) was used. Gene set enrichment analysis (GSEA) was performed, using the estimated protein fold changes to rank the proteins, with WebGestalt (http://webgestalt.org). The Gene Ontology (GO) classification for biological processes (GOBP) was used. The number of permutations was set to 1000. The IL-1β group was used to set a threshold for the “minimum number of genes for a category” in the conducted GSEA. The same threshold of a minimum of 10 genes for a category was used for GSEA of all groups. In addition to GSEA the protein list was filtered for elements with a log2 fold change ≥ 1.5 and a significance level of 0.05 [40]. Proteins were divided into the six categories (1) ECM, (2) anabolic, (3) catabolic, (4) growth factors, (5) immune system, and (6) other, with information retrieved from UniProt. Interaction network analysis of upregulated proteins was performed using STRING (http://string-db.org) [41]. Active interactions included text mining, experiments, and databases. Line thickness was set to indicate confidence.

For the DNA content, metabolic activity, LDH measurement, and immunoassay, statistical analysis of all data was performed using GraphPad Prism version 6.00. Normality was tested with D’Agostino-Pearson omnibus normality test, Shapiro-Wilk normality test, and Kolmogorov-Smirnov test with Dallal-Lilliefor *P* value. For parametric data, one-way ANOVA was performed. For non-parametric data, Kruskal-Wallis test was performed; *P* < 0.05 was regarded as significant.

## Results

### Viability of MSCs following priming with intervertebral disc conditioned medium

To investigate whether MSC viability was affected by the IVD CM or a proinflammatory environment, a cytotoxicity assay was performed (LDH), the DNA content and the metabolic activity were analyzed, and the cell morphology assessed. Measurements were performed at the end of the experiment following collection of MSC secretome. Stimulation of MSCs with healthy and degenerative IVD CM induced a significant increase in DNA content (1.78 ± 0.57 and 1.30 ± 0.27-fold, respectively) compared to the baseline condition (0.87 ± 0.24-fold) (*P* < 0.01), indicating enhanced cell proliferation. The traumatic stimulation induced a slight, although not significant, increase in DNA content (*P* = 0.241; 1.17 ± 0.26-fold). The DNA content following proinflammatory stimulus with IL-1β was similar to the baseline control (0.96 ± 0.30-fold, all normalized to day 0 after cell attachment) (Suppl. Fig. 2A).

Metabolic activity of MSCs following stimulation with all the IVD CM was increased compared to baseline control (Suppl. Fig. 2B). No significant changes were observed between different sources of IVD CM (*P* > 0.05) (healthy/traumatic/degenerative). LDH measurement in the MSC secretome showed no cytotoxicity in any culture condition (Suppl. Fig. 2C). Morphological analyses of MSCs supported findings from the metabolic and cytotoxicity assays; stimulation with healthy, traumatic, and degenerative IVD CM maintained a flattened cell morphology (Suppl. Fig. 2D).

### Proteins identified in MSC secretome and identification of enriched biological processes (GSEA)

Secretomes of MSCs treated with healthy, traumatic, degenerative IVD CM, or IL-1β were compared to the secretome of MSCs incubated in basal medium (baseline control). Compared to the baseline control, there were 224 significantly up- or downregulated proteins in MSC secretomes following healthy (Suppl. Table 1), 179 following traumatic (Suppl. Table 2), 223 following degenerative IVD CM (Suppl. Table 3), and 160 proteins following IL-1β stimulus (Suppl. Table 4) (all comparisons as fold changes relative to the baseline control).

**Fig. 2:**
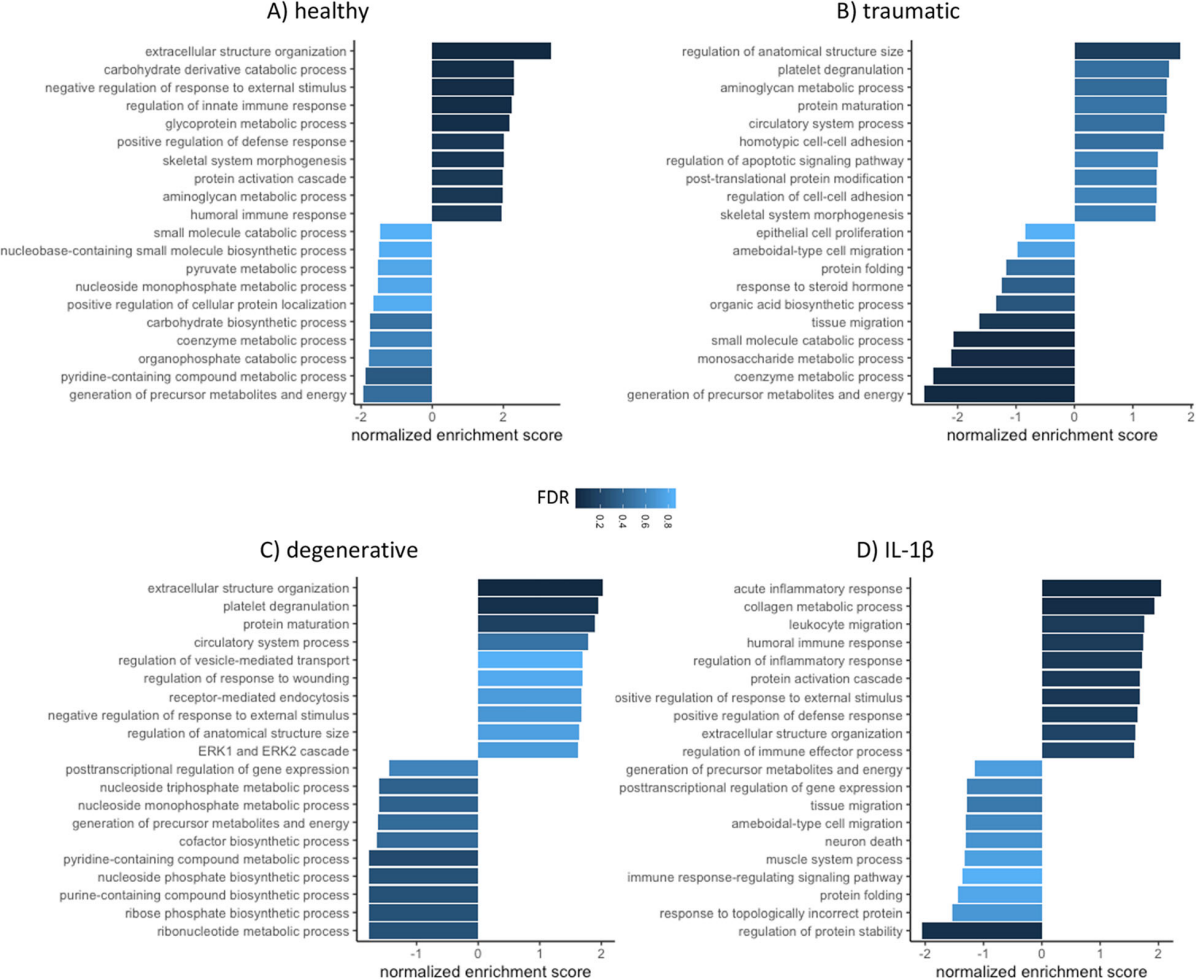
MSC secretome gene set enrichment analysis (GSEA). GSEA from LC-MS/MS data of released proteins following **a** healthy, **b** traumatic, **c** degenerative, and **d** IL-1β stimulus relative to baseline control. Figures display top 10 upregulated and downregulated gene ontology terms (functional database: biological processes). All reported GO terms have a *P* < 0.01. The false discovery rate (FDR) is color-coded with darker color indicating a low FDR

**Fig. 3:**
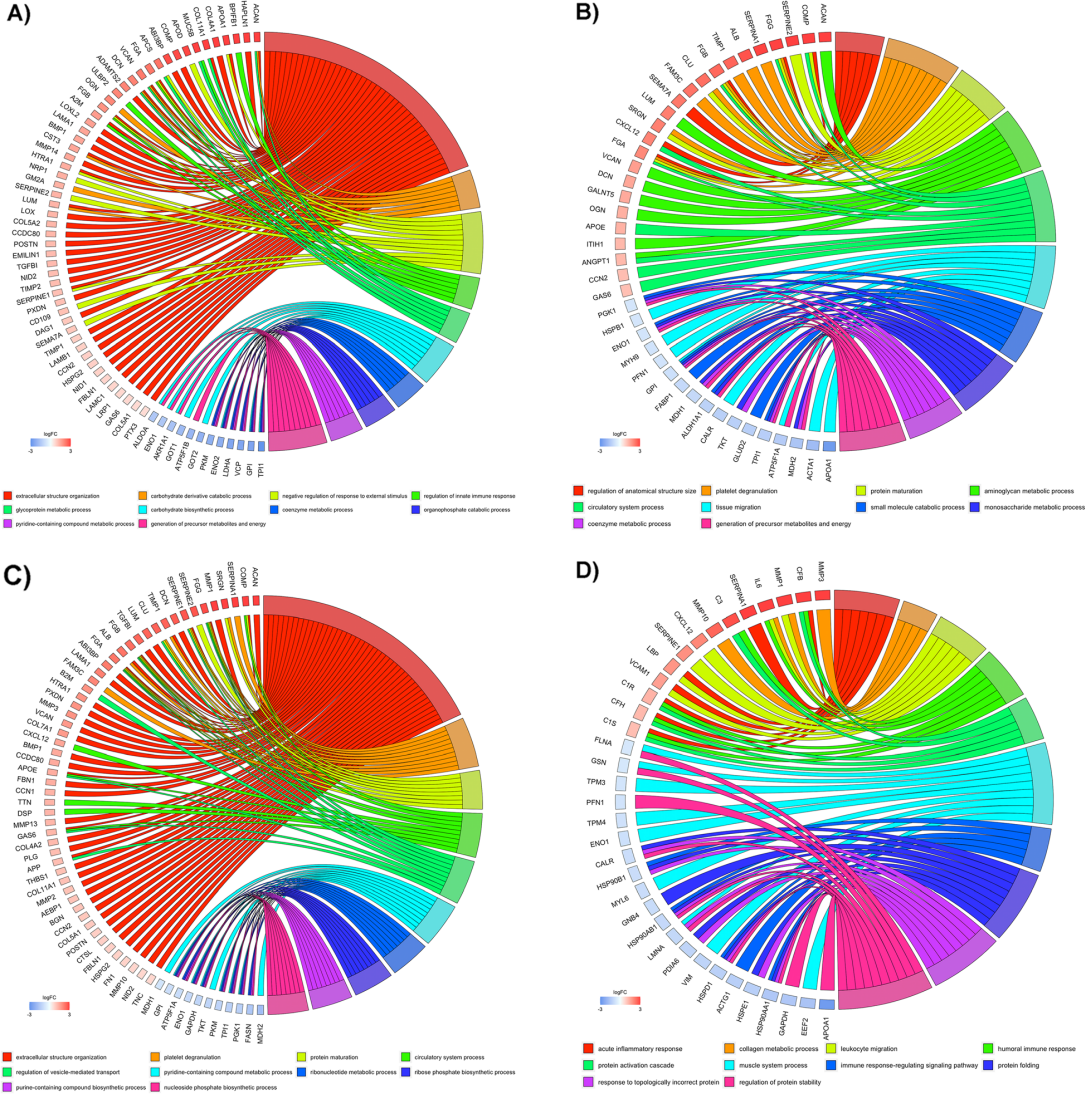
Gene set enrichment analysis (GSEA). Chord diagrams of top 5 upregulated and downregulated gene ontology (GO) terms (functional database: biological processes) found in **a** MSC “healthy” secretome gene set enrichment analysis; **b** MSC “traumatic” secretome gene set enrichment analysis; **c** MSC “degenerative” secretome gene set enrichment analysis; **d** MSC “IL-1β” secretome gene set enrichment analysis (LC-MS/MS data). All reported GO terms (right hemisphere) were significantly up- or downregulated (*P* < 0.01). Gene-box color (left hemisphere) indicates detection level as log2 fold change relative to the baseline control: upregulated detection = red, downregulated = blue. Strings indicate involvement of each gene in all respective GO-terms

Enriched biological processes (GO terms) were identified based on the GO classification system (GOBP). To allow for a comparison of the different experimental groups (healthy, traumatic, degenerative, IL-1β), all data were normalized to the respective MSC donor baseline control prior analysis. Within the significantly upregulated processes (*P* < 0.05), the identified GSEA terms with a false discovery rate (FDR) < 0.05 are listed in Fig. 2. MSC secretome following stimulation with healthy IVD CM showed an upregulation of extracellular structure organization (normalized enrichment score (NES) = 3.34, FDR = 0), carbohydrate derivative catabolic process (NES = 2.29, FDR = 0.007), negative regulation of response to external stimulus (NES = 2.28, FDR = 0.004), regulation of innate immune response (NES = 2.23, FDR = 0.006), glycoprotein metabolic process (NES = 2.1, FDR = 0.001), positive regulation of defense response (NES = 2.01, FDR = 0.029), skeletal system morphogenesis (NES = 2.01, FDR = 0.025), protein activation cascade (NES = 1.98, FDR = 0.028), aminoglycan metabolic process (NES = 1.98, FDR = 0.026), and humoral immune response (NES = 1.93, FDR = 0.03). Following stimulation with traumatic IVD CM, downregulation was observed for: small molecule catabolic process (NES = − 2.08, FDR = 0.006), monosaccharide metabolic process (NES = − 2.11, FDR = 0.009), coenzyme metabolic process (NES = − 2.41, FDR = 0.001), and generation of precursor metabolites and energy (NES = − 2.57, FDR = 0); while regulation of anatomical structure size was upregulated. Analyzing the secretome following stimulation with degenerative IVD CM induced upregulation of extracellular structure organization (NES = 2.03, FDR = 0.035), and platelet degranulation (NES = 1.95, FDR = 0.047). The proinflammatory exposure to IL-1β resulted in an upregulation of acute inflammatory response (NES = 2.05, FDR = 0.003) and collagen metabolic process (NES = 1.94, FDR = 0.019), while a significant downregulation for the regulation of protein stability (NES = − 2.05, FDR = 0.044) was observed. Interestingly, only two significantly upregulated biological processes were observed among multiple groups, namely “extracellular structure organization” (healthy, degenerative, and IL-1β), and “aminoglycan metabolic process” (healthy and traumatic). Proteins involved in the top five significantly upregulated and downregulated biological processes are displayed within a chord diagram in Fig. 3a–d.

**Fig. 4:**
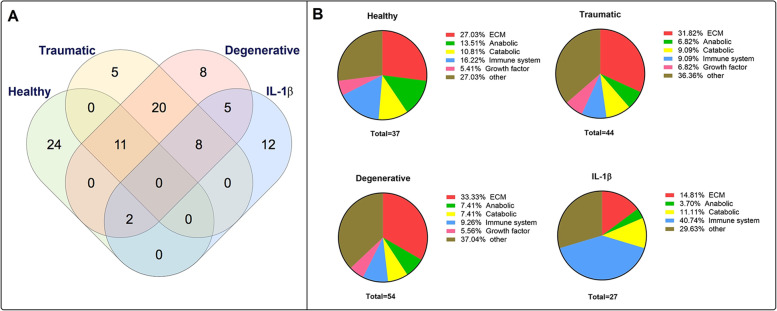
Comparison of secretome composition. **a** Venn-diagram representing the overlap of the detected proteins among all four experimental groups. **b** Pie charts representing MSC secretome compositions, categorized into anabolic, catabolic, ECM, growth factor, immune system, and other proteins. Data includes the detected proteins (LC-MS/MS data) upregulated > 1.5 log2 fold relative to the baseline control

**Fig. 5:**
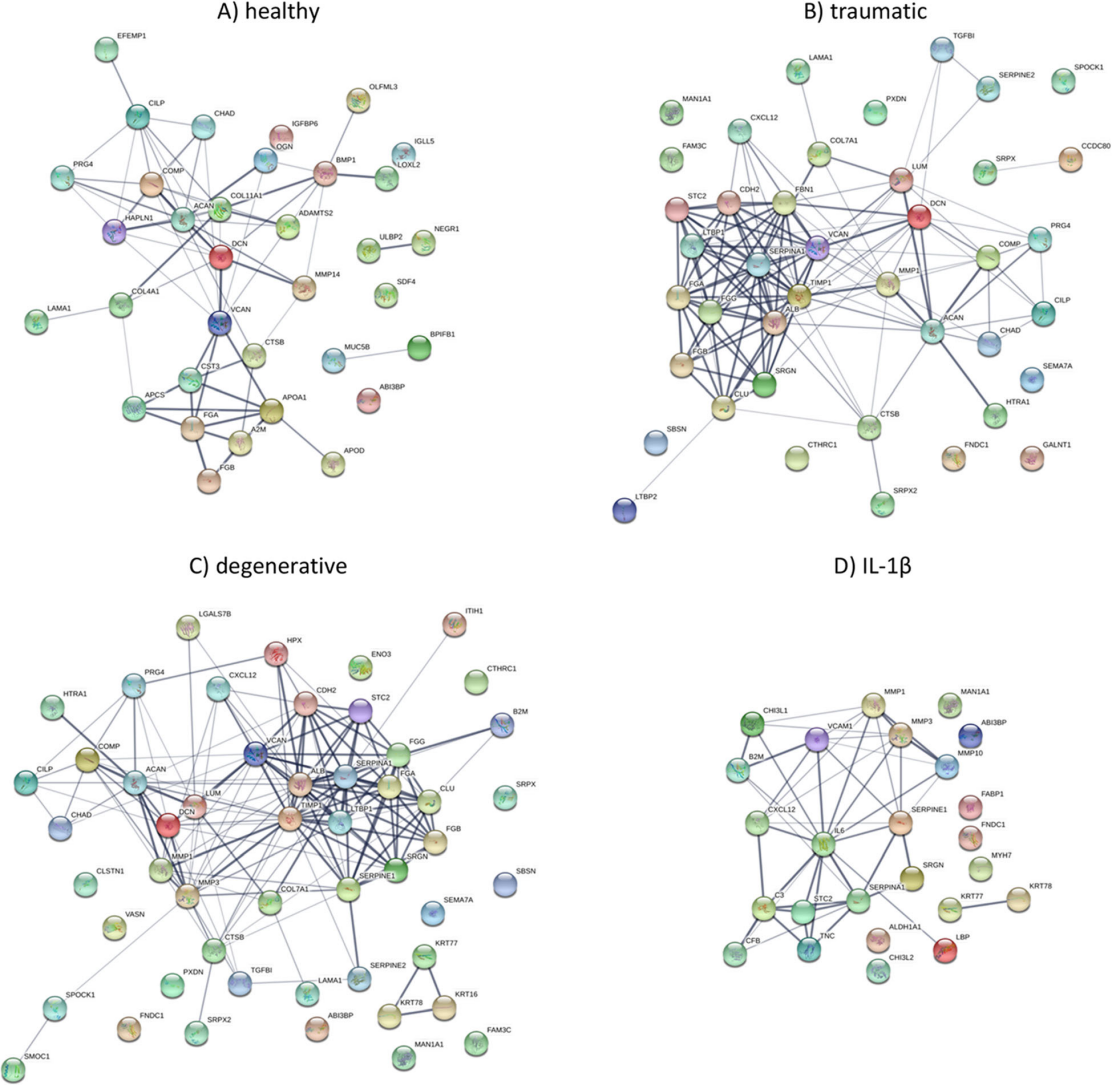
Interaction network of upregulated proteins in MSC secretome. Data includes detected proteins (LC-MS/MS data) upregulated > 1.5 log2 fold relative to baseline control. Upregulated proteins of **a** healthy, **b** traumatic, **c** degenerative, and **d** IL-1β stimulated MSC secretomes have been searched for possible interaction with the network analysis of String. Active interactions include text mining, experiments, and databases. Line thickness indicates confidence. Proteins are labeled with the corresponding gene name

Proteins with a log2 fold-change > 1.5 (relative to the baseline) were further compared among the 4 experimental groups (Table 1). The most pronounced overlap in secreted proteins was found between the MSC secretomes following traumatic and degenerative stimulation (38 proteins). These two groups shared 11 (traumatic) and 13 (degenerative) secreted proteins with the secretome of MSCs stimulated by healthy CM (Fig. 4a). The highest overlap with the proinflammatory control was found in the secretome of MSCs stimulated with degenerative CM (14 proteins) followed by traumatic (8 proteins) and healthy (2 proteins) CM stimulation (Fig. 4a). Proteins were further categorized into anabolic, catabolic, ECM, growth factor, immune system, or other proteins (Fig. 4b). Of note, the percentage of secreted factors associated with immune response was almost doubled following exposure of MSCs to a healthy IVD CM environment (16%) compared to a traumatic or degenerative IVD CM stimulus (average: 9%) and was highest after MSC stimulation with IL-1β (41%). Network analysis of STRING revealed possible interactions among the upregulated proteins of healthy (Fig. 5a), traumatic (Fig. 5b), degenerative (Fig. 5c) CM, and IL-1β-induced MSC secretomes (Fig. 5d).

**Table 1:** Proteins identified in secretomes of mesenchymal stromal cells stimulated with healthy, traumatic, or degenerative intervertebral disc conditioned medium or IL1-β, with a log2 fold-change > 1.5 (relative to the baseline control stimulation)

gene_ID	protein_ID	Medium stimulus
CHAD	O15335	Healthy, Traumatic, Degenerative
CILP	O75339	Healthy, Traumatic, Degenerative
FGA	P02671	Healthy, Traumatic, Degenerative
FGB	P02675	Healthy, Traumatic, Degenerative
DCN	P07585	Healthy, Traumatic, Degenerative
CTSB	P07858	Healthy, Traumatic, Degenerative
VCAN	P13611	Healthy, Traumatic, Degenerative
ACAN	P16112	Healthy, Traumatic, Degenerative
LAMA1	P25391	Healthy, Traumatic, Degenerative
COMP	P49747	Healthy, Traumatic, Degenerative
PRG4	Q92954	Healthy, Traumatic, Degenerative
IGHA1	P01876	Healthy, Degenerative, IL-1β
ABI3BP	Q7Z7G0	Healthy, Degenerative, IL-1β
STC2	O76061	Traumatic, Degenerative, IL-1β
SERPINA1	P01009	Traumatic, Degenerative, IL-1β
MMP1	P03956	Traumatic, Degenerative, IL-1β
SRGN	P10124	Traumatic, Degenerative, IL-1β
MAN1A1	P33908	Traumatic, Degenerative, IL-1β
CXCL12	P48061	Traumatic, Degenerative, L-1β
B2M	P61769	Traumatic, Degenerative, IL-1β
FNDC1	Q4ZHG4	Traumatic, Degenerative, IL-1β
SRPX2	O60687	Traumatic, Degenerative
SEMA7A	O75326	Traumatic, Degenerative
TIMP1	P01033	Traumatic, Degenerative
FGG	P02679	Traumatic, Degenerative
ALB	P02768	Traumatic, Degenerative
SERPINE2	P07093	Traumatic, Degenerative
CLU	P10909	Traumatic, Degenerative
CDH2	P19022	Traumatic, Degenerative
LUM	P51884	Traumatic, Degenerative
SRPX	P78539	Traumatic, Degenerative
COL7A1	Q02388	Traumatic, Degenerative
SPOCK1	Q08629	Traumatic, Degenerative
LTBP1	Q14766	Traumatic, Degenerative
TGFBI	Q15582	Traumatic, Degenerative
SBSN	Q6UWP8	Traumatic, Degenerative
FAM3C	Q92520	Traumatic, Degenerative
PXDN	Q92626	Traumatic, Degenerative
HTRA1	Q92743	Traumatic, Degenerative
CTHRC1	Q96CG8	Traumatic, Degenerative
HAPLN1	P10915	Healthy
BPIFB1	Q8TDL5	Healthy
APOA1	P02647	Healthy
COL4A1	P02462	Healthy
COL11A1	P12107	Healthy
MUC5B	Q9HC84	Healthy
IGKC	P01834	Healthy
APOD	P05090	Healthy
IGHG1	P01857	Healthy
APCS	P02743	Healthy
IGLL5	B9A064	Healthy
ADAMTS2	O95450	Healthy
ULBP2	Q9BZM5	Healthy
OGN	P20774	Healthy
EFEMP1	Q12805	Healthy
IGFBP6	P24592	Healthy
OLFML3	Q9NRN5	Healthy
A2M	P01023	Healthy
LOXL2	Q9Y4K0	Healthy
NEGR1	Q7Z3B1	Healthy
SDF4	Q9BRK5	Healthy
BMP1	P13497	Healthy
CST3	P01034	Healthy
MMP14	P50281	Healthy
FBN1	P35555	Traumatic
GALNT1	Q10472	Traumatic
CCDC80	Q76M96	Traumatic
LTBP2	Q14767	Traumatic
HPX	P02790	Degenerative
ITIH1	P19827	Degenerative
LGALS7; LGALS7B	P47929	Degenerative
CLSTN1	O94985	Degenerative
SMOC1	Q9H4F8	Degenerative
ENO3	P13929	Degenerative
VASN	Q6EMK4	Degenerative
KRT16	P08779	Degenerative
CFB	P00751	IL1-β
IL6	P05231	IL1-β
C3	P01024	IL1-β
MMP10	P09238	IL1-β
MYH7	P12883	IL1-β
ALDH1A1	P00352	IL1-β
LBP	P18428	IL1-β
FABP1	P07148	IL1-β
CHI3L1	P36222	IL1-β
VCAM1	P19320	IL1-β
CHI3L2	Q15782	IL1-β
TNC	P24821	IL1-β

### Quantitative measurement of protein concentration by multiplex immunoassay

**Fig. 6:**
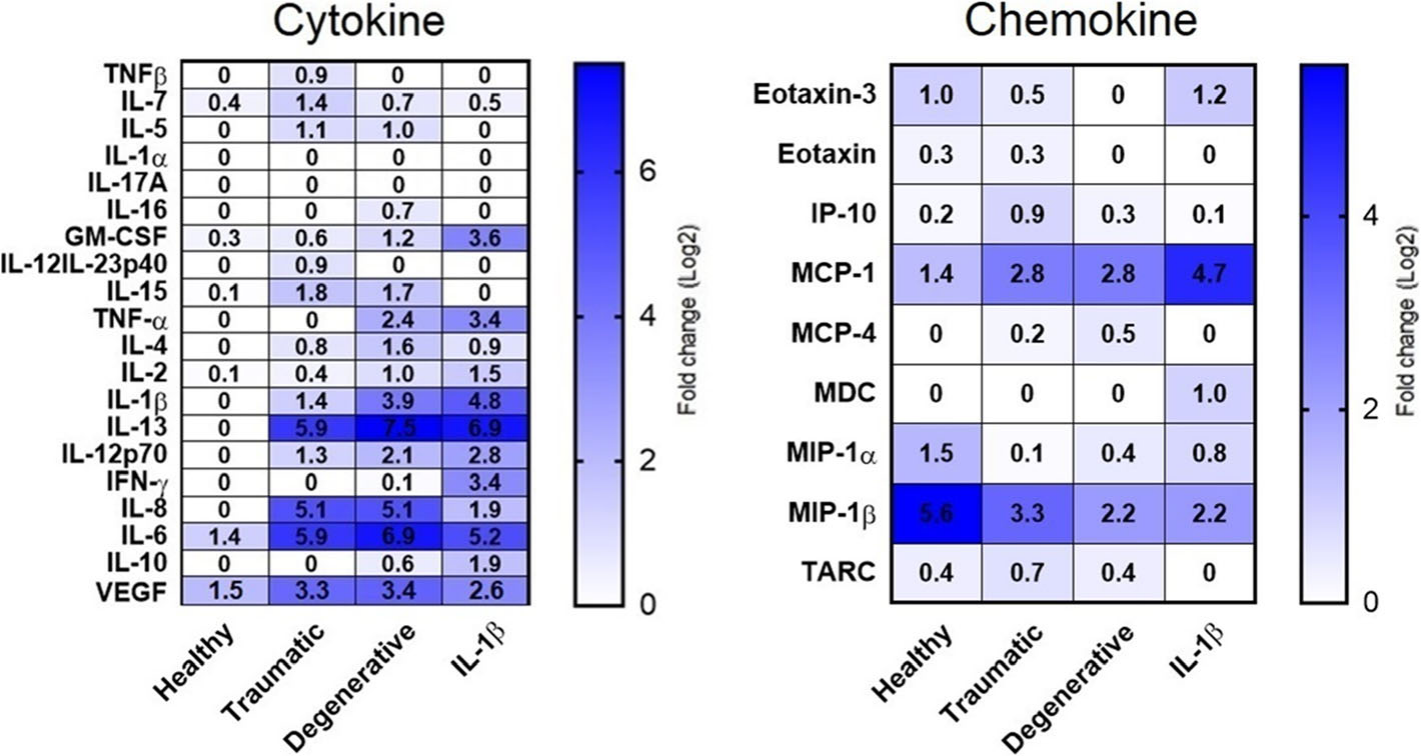
Immunoassay data of MSC secretomes. Cytokines and chemokines quantified in two immunoassay panels. Data includes quantified proteins as log2 fold-change relative to baseline control. Darker color indicates a higher upregulation. 0 = not detected

Levels of different cytokines and chemokines were measured in pooled IVD CM (Suppl. Table 5), and in secretomes of MSCs following culture in basal medium (baseline control) and following exposure to IVD CMs (healthy, traumatic, degenerative) and IL-1β (*N*  =  9/ group). In MSC secretomes, IL-1α and IL-17A were either undetected or extremely low in all samples and could not be analyzed (Fig. 6). Interestingly, most of the proinflammatory and/or immunomodulatory cytokines were undetected in the secretomes of MSCs incubated with healthy IVD CM, including TNF-β, IL-5, IL-16, IL-12, IL-23p40, TNF-α, IL-4, IL-1β, IL-13, IL-12p70, IFN-γ, IL-8, and IL-10. In contrast, the concentration of several chemokines/cytokines such as GM-CSF, TNF-α, IL-12p70, IFN-γ (all *P* < 0.05), monocyte chemoattractant protein 1 (MCP-1), macrophage inflammatory protein 1 beta (MIP-1β), VEGF, IL-13, and IL-6 (all *P* < 0.01) was significantly higher in the secretomes of IL-1β primed MSCs than those of baseline control. There was a significant difference between traumatic IVD CM group and baseline control regarding the fold changes of IL-13, IL-8, IL-6 (*P* < 0.01), VEGF, MCP-1, and MIP-1β (*P* < 0.05). Moreover, the concentration of IL-13, IL-8, IL-6 (*P* < 0.01), TNF-α, IL-1β, VEGF, MCP-1, and MIP-1β (fold changes *P* < 0.05) was significantly higher in secretomes of MSCs exposed to degenerative IVD CM than the baseline control group. In both traumatic and degenerative IVD CM groups, a similar trend was observed for production of cytokines, including IL-13, IL-8, and IL-6 which were considerably higher than the healthy group (*P* < 0.01). On the other hand, the secretion of MIP-1β, a chemoattractant for inflammatory cells, was considerably higher in MSC secretome following stimulation by healthy IVD CM in comparison to other treatment groups (*P* < 0.05).

## Discussion

The present study aimed to investigate the response of MSCs following exposure to a healthy, traumatic, or degenerative human IVD environment. Our primary finding was that stimulation with IVD CM induced a richer and more complex MSC secretome, involving more biological processes, compared to stimulation with a single cytokine (IL-1β). Secondly, the MSCs response to stimulation with IVD CM was different based on their status (i.e., healthy versus traumatic/degenerated). Thirdly, the MSC secretome seemed to match the primary need of the IVD tissue: homeostasis maintenance in the case of healthy IVDs, immunomodulation, adjustment of the matrix synthesis/degradation disbalance, and (re) organization of ECM in the case of traumatic and degenerative IVDs.

When exposed to a healthy IVD CM, MSCs response reflected the maintenance of homeostasis. This was expected, as in the absence of injury or degeneration, there is no need for tissue repair or regeneration. Successful IVD homeostasis involves a well-regulated, low dose of immunomodulatory cytokines and chemokines [42]. GSEA of healthy IVD-induced MSC secretome revealed enriched “regulation of innate immune response” and “humoral immune response.” Interestingly, the proportion of secreted proteins associated with the immune system was also higher following stimulation by healthy IVD CM compared to the traumatic and degenerative stimulation. For example, upregulation of Amyloid P was observed exclusively after the healthy stimulus (Table 1). Amyloid P inhibits the differentiation of fibrocytes, promotes the formation of immuno-regulatory macrophages, and inhibits neutrophil adhesion to ECM [43]. Immunoassay data indicated a significantly increased release of MIPs following exposure to a healthy environment. MIP-1β represents a ligand of C-C chemokine receptor type 5 (CCR5), which has been identified on IVD cells and may be involved in cell migration [44]. MIP-1α has been associated with inhibition of cell proliferation [45]. Release of MIP-1α might therefore represent an autoregulation of cell proliferation, as the intact healthy environment does not require an enriched cell population [46, 47]. Concerning ECM turnover, we observed an upregulated release of alpha 2 macroglobulin (A2M) (Table 1). A2M represents the major inhibitor of endoproteinases and its release might indicate that MSCs aim to stabilize the healthy IVD ECM in its physiological composition and structure.

The IVD depends on an intact ECM to maintain its primary mechanical function. Damage following traumatic injury represents a serious threat to the integrity of IVD ECM. Exposure of MSCs to traumatic IVD CM enriched the process “regulation of anatomical structure size.” MSCs secreted several proteins solely following traumatic stimulus, potentially participating in damage repair. Latent transforming growth factor-β1 binding protein 2 (LTBP-2, Table 1) has been reported to stimulate the expression of TGF-β1 in fibroblasts [48]. In the IVD, TGF-β1 has been associated with alleviation of the inflammatory response, inhibition of ECM degradation, inhibition of cell death, increased ECM synthesis, and promotion of cell proliferation [49]. Similarly, we observed an upregulation of cellular communication network factor 2/connective tissue growth factor (CCN2/CTGF) mainly following traumatic stimulus (Suppl. Table 2). CCN2/CTGF, also secreted by notochordal cells [50], can induce matrix production in nucleus pulposus (NP) cells [51]. After traumatic IVD CM stimulation, there were also upregulated levels of angiopoietin 1 (Suppl. Table 2), which represents the ligand of angiopoietin 1 receptor (Tie2) and is crucial for the survival of NP cells [52]. The overall MSCs response to traumatic IVD CM thus points towards cell growth and ECM repair.

In contrast to the immediate threat of ECM damage in traumatic IVDs, degeneration is a process evolving over decades, eventually resulting in a highly disorganized, fragmented ECM. As IVD degeneration is often associated with ingrowth of nerves and blood vessels, neural and vascular cells may have contributed to the composition of the degenerative IVD CM. Exposure of MSCs to a degenerative IVD environment did not enhance any immunomodulatory biological processes. However, secretion of certain proteins holding immunomodulatory functions such as keratin 16 (KRT16) and galectin-7 (LGALS7) was solely induced following degenerative stimulus (Table 1) [53, 54]. Concerning tissue regeneration, secreted modular calcium-binding protein 1 (SMOC-1, Table 1) was upregulated, which has been associated with TGF-β1 mediated fibrosis [55]. Interestingly, while the upregulated biological processes did differ between the four experimental groups, we did not observe any relevant differences in the downregulated processes (Fig. 2). Nevertheless, downregulation or absence of certain proteins might also be of importance for secretome mediated influence on biological processes.

Among all groups, the highest overlap in released proteins was observed following stimulation by traumatic and degenerative IVD CM. Both traumatic and degenerative stimuli induced secretion of matrix metalloproteinase (MMP)1, MMP2, MMP10, and MMP13 (Suppl. Table 2/3). MMPs represent matrix-degrading enzymes which can facilitate migration and recruitment of inflammatory cells or progenitor cells. Furthermore, MSCs are known to secrete MMPs to be able to migrate through the ECM [56]. Tissue inhibitors of metalloproteinases (TIMPs) are important MMP antagonists. They control MMP activities which results in a balanced matrix degradation. Interestingly, TIMP1 was upregulated following traumatic and degenerative stimulus (Table 1). This suggests that MSCs try to stabilize a dynamic balance of ECM turnover during reorganization following trauma or during regeneration.

MSCs also released interleukins (IL-1, IL-6, IL-8, IL-13), serglycin (SRGN), and complement component 1r following traumatic and degenerative IVD CM stimulus (Suppl. Table 2/3). IL-1β triggers the innate inflammation while IL-8 induces chemotaxis in neutrophils and MSCs, causing them to migrate towards the site of damage [57–59]. In contrast, IL-13 inhibits the production of proinflammatory cytokines and chemokines [60]. SRGN represents a binding partner for various chemokines and functions as a modulator of the immune system [61]. The immunoassay also revealed a significantly higher concentration of IL-6 following traumatic and degenerative compared to a healthy stimulus. IL-6 represents a key player of the primary inflammatory response where it functions as an important activator of pro- and anti-inflammatory cells [62]. Thus, the secreted proteins may have a role in modulating the inflammatory state in acute and chronic IVD pathologies.

STRING analysis revealed denser networks following traumatic and degenerative IVD CM stimulation, when compared to the healthy stimulus. Interestingly, the common center of both networks was alpha-1-antitrypsin, or SERPINA1, a broad-spectrum protease inhibitor that was associated with the regulation of inflammatory responses [63]. For the IVD, we can only speculate about the role of SERPINA1. Potential targets include its inhibition of proteinase 3 (PRTN3), MMP9, and a disintegrin and metalloprotease domain 17 (ADAM17), among others [64]. High levels of PRTN3 have been associated with calcifications in herniated IVD tissue [65]. Upregulated expression of MMP9 has been reported in cells from degenerative compared to traumatic IVDs [66]. Similarly, an increased concentration in MMP9 has been correlated with the degree of degeneration and with pain sensation in herniated IVD [66, 67]. Inhibition of ADAM17 has been associated with an upregulated TGFβ signaling [68]. In our data, degenerative IVD CM induced a higher release of SERPINA1 compared to traumatic stimulus, while healthy CM did not provoke SERPINA1 secretion. This might indicate that MSCs participate in the restoration of protease inhibitors known to deplete during IVD degeneration [69].

To validate the MSCs response, we also performed a simplistic stimulation with IL-1β. This resulted in enriched biological processes like “acute inflammatory response”, “regulation of inflammatory response,” and “leukocyte migration.” Also, IL-1β stimulus led to the secretion of various proinflammatory factors including elements of the complement system, TNF-α, IL-1β, and IL-6 (Suppl. Table 4). Elevated levels of TNF-α and IL-6 have previously been observed in MSCs following exposure to IL-1β [70]. Quantitative immunoassay revealed significantly higher concentrations of MCP-1 following IL-1β stimulus compared to stimulation by healthy IVD CM. MCP-1 contributes to the activation and recruitment of monocytes and has been detected in normoxic and hypoxic cultured MSCs [71].

The present study was performed using a 2D monolayer cell culture model. 3D culture systems require embedding MSCs within a carrier material (e.g., hydrogel) which might bind some of the released proteins and therefore alter the profile of proteins secreted by the cell-hydrogel construct. In addition, LC-MS/MS is very sensitive, and proteins released from the carrier material could potentially mask proteins released as part of the MSC secretome. However, it has been reported that MSC secretome composition may change following MSC culture within a 3D environment. For example, concentrations of hepatocyte growth factor (HGF) and intercellular adhesion molecule 1 (ICAM-1) were increased in 3D cultures compared to 2D cultures [29]. Furthermore, 3D culture of umbilical cord tissue MSCs has been associated with an enhanced expression of anti-inflammatory cytokines (IL-10 and LIF) and trophic factors involved in mechanisms leading to tissue regeneration compared to the secretome following 2D culture [72]. Further study should investigate the response of MSCs cultured within a 3D system to the different IVD environments. Besides, we investigated the composition of the MSC secretome at 24–48 h after stimulation. While this time frame is of relevance for short-term MSC preconditioning for secretome generation, it only partially reflects the situation occurring after therapeutic MSC injection into the IVD. In an in vivo or ex-vivo environment, it is likely that the MSC secretome may change over time and depending on the surrounding conditions.

As MSCs have been shown to have diverse properties based on age and gender, we analyzed whether the secretome compositions were different between young versus old and male versus female donors [73]. Interestingly, we neither observed any significant differences in secretome composition among the different donor age groups nor between female or male donors. This might indicate that the stimulus applied to the MSCs was more prominent to generate a certain type of secretome than the MSC source.

### Limitations

Here we analyzed the molecular factors and the biological processes within the MSC secretome based on protein identification. Besides soluble proteins, secretome contains free nucleic acids, lipids, and extracellular vesicles. Our results therefore only reflect a portion of the MSC secretome. Secondly, IVD CM contains high amounts of ECM proteins, which potentially mask proteins present at low concentrations during LC-MS/MS. We therefore replaced the CM following 24 h stimulation to collect the proteins secreted by MSCs. It is likely that MSCs already secreted proteins during the first 24 h of stimulation by IVD CM which were not detected with this experimental setup. Thirdly, we did not perform any co-culture of MSCs and IVD cells, as the proteins released by both cell types could not have been differentiated in the analysis process. However, the use of cell free IVD conditioned medium omits the possible changes in secretome composition evolving from cell-cell or cell-ECM contact. Finally, we were not able to assign the degree of degeneration to IVDs from all donors since magnetic resonance imaging (MRI) scans were only available from donors with degenerative IVDs.

## Conclusions

Our results indicate that MSCs adapt their profile of secreted proteins depending on their environment. This allows them to take different roles tailored to the state of disease. In healthy IVDs, MSC-derived trophic factors may stabilize tissue homeostasis and act as immunomodulators. Following a traumatic injury, MSC secretome may support the recruitment of additional cells, modulate inflammation, cell survival, and secrete ECM proteins. A degenerative IVD milieu may induce factors initiating remodeling processes and synthesis of ECM proteins. However, characterization of the proteins released by MSCs represents only one part of the interaction between MSCs and the IVD milieu. The response of the resident IVD cells to the MSC secretome is currently under investigation and will provide important knowledge to identify the therapeutic secretome for specific IVD states of injury or degeneration.

## Supplementary Information

**Additional file 1: Supplementary Figure 1**. Details of cell and tissue samples used for the experiments. (**A)** MSCs from twelve different donors were used. All MSCs were derived from vertebral bone marrow aspirates. Only donors younger than 50 years (age at isolation) were selected, representing four different age groups (average age 17, 26.33, 37.66 and 48.66 years). Gender was equally balanced (6 male; 6 female) and symmetrically distributed among age groups. **(B)** IVD conditioned medium donor overview. For MSC stimulation, IVD conditioned medium from different donors within one condition was pooled (*n* = 4/group).

**Additional file 2: Supplementary Figure 2**. Effect of IVD conditioned medium treatment on DNA content, metabolic activity and lactate dehydrogenase (LDH) release of MSCs. **(A)** DNA content of MSCs in 6-well plate normalized to timepoint zero after 14 h of cell attachment. *p < 0.05, ***p < 0.001 (Kruskal-Wallis test). **(B)** Metabolic activity was measured with CellTiter-Blue. Data was standardized to the treatment condition baseline within every MSC donor. *p < 0.05, **p < 0.01, ***p < 0.001, ****p < 0.0001; One-way ANOVA. **(C)** LDH was measured in the MSC secretome to detect cytotoxic reactions. No significant differences were found (Kruskal-Wallis-test). **(D-H)** Images were taken just before secretome collection. Scale bar = 500 μm.

**Additional file 3 : Supplementary Table 1.** MSC secretome following healthy CM stimulation. **Supplementary Table 2.** MSC secretome following traumatic CM stimulation. **Supplementary Table 3.** MSC secretome following degenerative CM stimulation. **Supplementary Table 4.** MSC secretome following IL-1β stimulation.

**Additional file 4 : Supplementary Table 5** Concentrations of cytokines and chemokines in pooled conditioned media from healthy, traumatic and degenerative intervertebral disc, measured by immunoassay technique (mean+/-sd of technical replicates; pg/mL).

## Data Availability

Proteomics data are reported in the supplementary tables of the manuscript. All original data are available from the authors on request. The mass spectrometry proteomics data have been deposited to the ProteomeXchange Consortium via the PRIDE partner repository with the dataset identifier PXD021281 [74].

## Abbreviations

A2M: Alpha 2 macroglobulin
ADAM: A disintegrin and metalloprotease domain
a-MEM: Alpha minimal essential medium
ASC: Adipose-derived stem cells
CCN2: Cellular communication network factor 2
CCR5: C-C chemokine receptor type 5
CM: Conditioned medium
CTGF: Connective tissue growth factor
ECM: Extracellular matrix
FBS: Fetal bovine serum
FDR: False discovery rate
FGF: Fibroblast growth factor
G-CSF: Granulocyte colony-stimulating factor
GO: Gene ontology
GOBP: Gene ontology classification for biological processes
GSEA: Gene set enrichment analysis
HGF: Hepatocyte growth factor
IGF-1: Insulin-like growth factor 1
IL: Interleukin
IL1-Ra: Interleukin 1 receptor antagonist
INF: Interferon
ISSCR: International society for stem cell research
IVD: Intervertebral disc
KRT16: Keratin 16
LC-MS/MS: Liquid chromatography mass spectroscopy/ mass spectroscopy
LDH: Lactate dehydrogenase
LGALS7: Galectin 7
Lg-DMEM: Low glucose Dulbecco’s modified Eagle’s medium
LTBP-2: Latent transforming growth factor-β1 binding protein 2
MCP: Monocyte chemoattractant protein
MIP: Macrophage inflammatory protein
MMP: Matrix metalloproteinase
MRI: Magnetic resonance imaging
MSC: Mesenchymal stromal cells
NES: Normalized enrichment score
NP: Nucleus pulposus
P/S: Penicillin-Streptomycin
PBS: Phosphate buffered saline
PRTN3: Proteinase 3
RT: Room temperature
SMOC-1: Secreted modular calcium-binding protein 1
SRGN: Serglycin
TIMP: Tissue inhibitor of metalloproteinase
TNF: Tumor necrosis factor
VEGF: Vascular endothelial growth factor
