# Supplementary figures and images for: Uncovering the secretome of mesenchymal stromal cells exposed to healthy, traumatic, and degenerative intervertebral discs: a proteomic analysis
Source: Stem Cell Res Ther. 2021 Jan 7;12:11. doi: 10.1186/s13287-020-02062-2 (PMC7789679; doi:10.1186/s13287-020-02062-2)

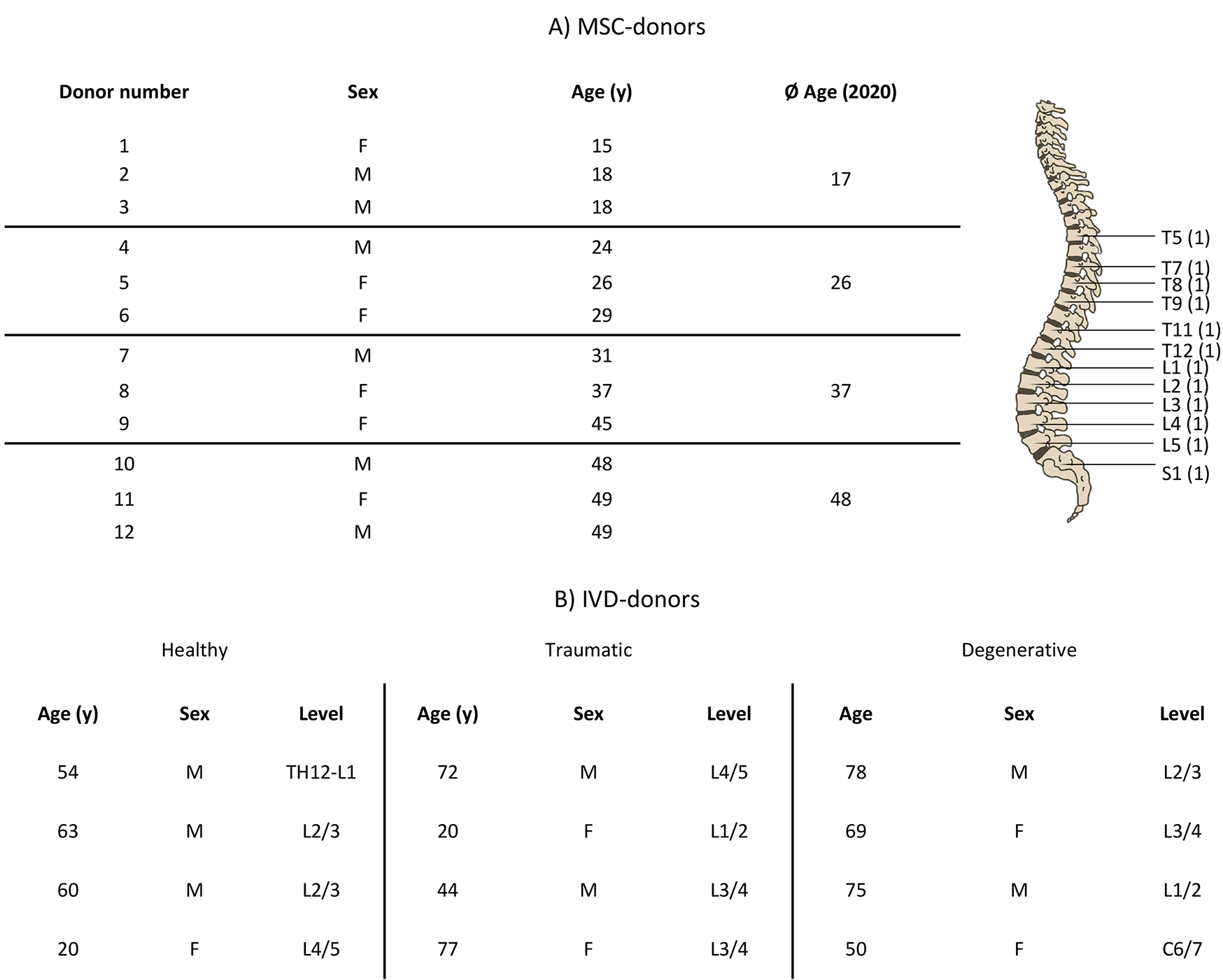

Supplement: Supplementary file 1 — Additional file 1: Supplementary Figure 1. Details of cell and tissue samples used for the experiments. (A) MSCs from twelve different donors were used. All MSCs were derived from vertebral bone marrow aspirates. Only donors younger than 50 years (age at isolation) were selected, representing four different age groups (average age 17, 26.33, 37.66 and 48.66 years). Gender was equally balanced (6 male; 6 female) and symmetrically distributed among age groups. (B) IVD conditioned medium donor overview. For MSC stimulation, IVD conditioned medium from different donors within one condition was pooled (n = 4/group). [file 13287_2020_2062_MOESM1_ESM.tif]

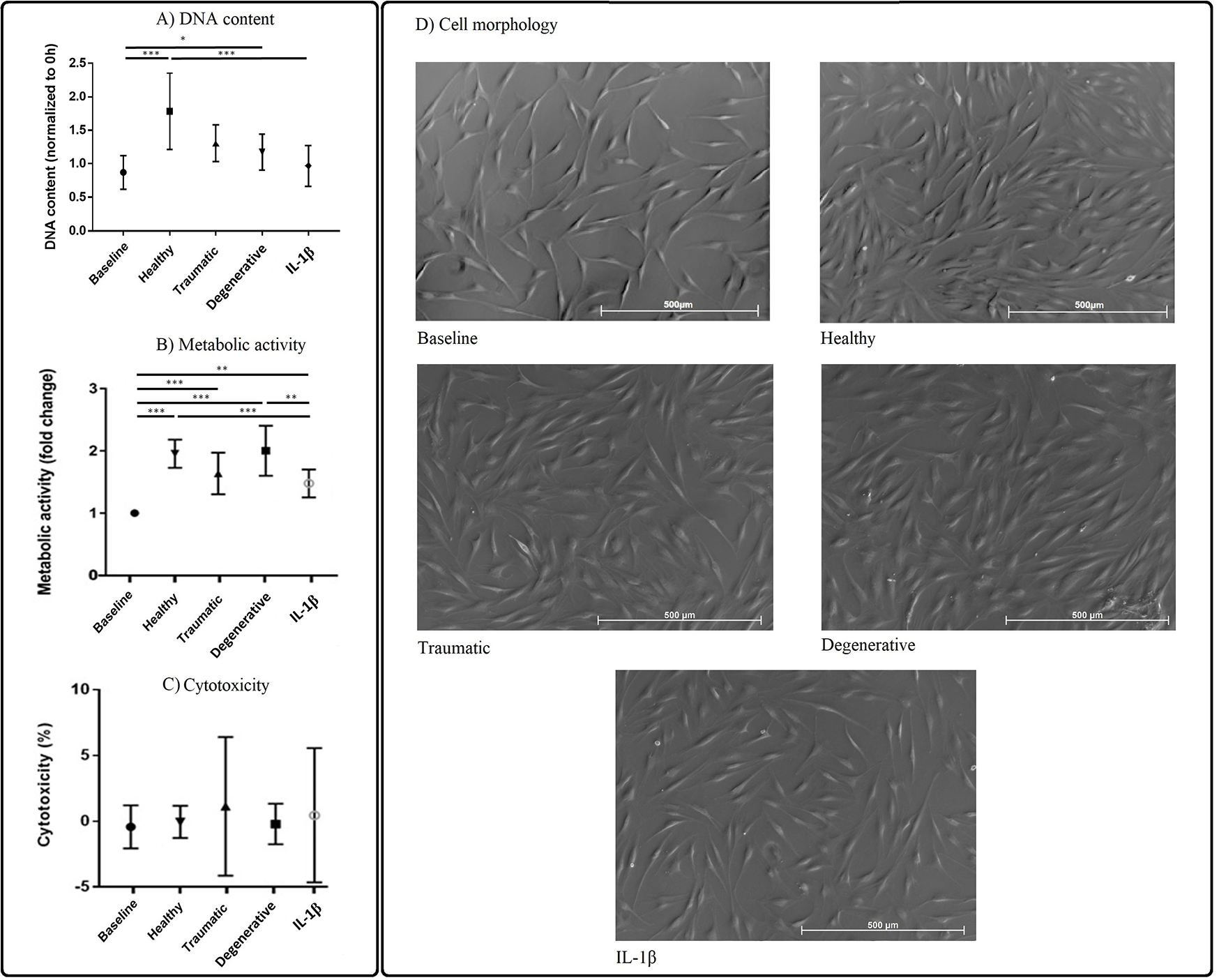

Supplement: Supplementary file 2 — Additional file 2: Supplementary Figure 2. Effect of IVD conditioned medium treatment on DNA content, metabolic activity and lactate dehydrogenase (LDH) release of MSCs. (A) DNA content of MSCs in 6-well plate normalized to timepoint zero after 14 h of cell attachment. *p < 0.05, ***p < 0.001 (Kruskal-Wallis test). (B) Metabolic activity was measured with CellTiter-Blue. Data was standardized to the treatment condition baseline within every MSC donor. *p < 0.05, **p < 0.01, ***p < 0.001, ****p < 0.0001; One-way ANOVA. (C) LDH was measured in the MSC secretome to detect cytotoxic reactions. No significant differences were found (Kruskal-Wallis-test). (D-H) Images were taken just before secretome collection. Scale bar = 500 μm. [file 13287_2020_2062_MOESM2_ESM.tif]
