# Supplementary material for: Uncovering the secretome of mesenchymal stromal cells exposed to healthy, traumatic, and degenerative intervertebral discs: a proteomic analysis
Source: Stem Cell Res Ther. 2021 Jan 7;12:11. doi: 10.1186/s13287-020-02062-2 (PMC7789679; doi:10.1186/s13287-020-02062-2)
Supplement: Supplementary file 3 — Additional file 3 : Supplementary Table 1. MSC secretome following healthy CM stimulation. Supplementary Table 2. MSC secretome following traumatic CM stimulation. Supplementary Table 3. MSC secretome following degenerative CM stimulation. Supplementary Table 4. MSC secretome following IL-1β stimulation. [file 13287_2020_2062_MOESM3_ESM.docx]

| **Supplementary Table 1.** MSC secretome following healthy CM stimulation | | | |
| --- | --- | --- | --- |
| **gene_ID** | **protein_ID** | **Fold change (Log2)** | **p.value.adjusted** |
| ACAN | P16112 | 6.08 | 0.000 |
| HAPLN1 | P10915 | 5.12 | 0.000 |
| CHAD | O15335 | 4.88 | 0.000 |
| BPIFB1 | Q8TDL5 | 4.14 | 0.023 |
| PRG4 | Q92954 | 4.11 | 0.028 |
| APOA1 | P02647 | 3.60 | 0.000 |
| COL4A1 | P02462 | 3.27 | 0.000 |
| COL11A1 | P12107 | 3.09 | 0.000 |
| MUC5B | Q9HC84 | 3.08 |  |
| IGKC | P01834 | 3.03 | 0.010 |
| CILP | O75339 | 3.00 |  |
| APOD | P05090 | 2.93 |  |
| COMP | P49747 | 2.89 | 0.004 |
| ABI3BP | Q7Z7G0 | 2.84 | 0.000 |
| IGHG1 | P01857 | 2.49 | 0.002 |
| APCS | P02743 | 2.34 |  |
| FGA | P02671 | 2.28 |  |
| IGLL5 | B9A064 | 2.24 |  |
| VCAN | P13611 | 2.23 | 0.000 |
| IGHA1 | P01876 | 2.18 | 0.008 |
| DCN | P07585 | 2.12 | 0.000 |
| ADAMTS2 | O95450 | 2.08 | 0.000 |
| ULBP2 | Q9BZM5 | 2.06 | 0.000 |
| OGN | P20774 | 2.04 |  |
| EFEMP1 | Q12805 | 1.90 | 0.000 |
| FGB | P02675 | 1.87 |  |
| IGFBP6 | P24592 | 1.85 | 0.023 |
| OLFML3 | Q9NRN5 | 1.72 | 0.007 |
| A2M | P01023 | 1.71 | 0.000 |
| CTSB | P07858 | 1.67 | 0.002 |
| LOXL2 | Q9Y4K0 | 1.63 | 0.000 |
| NEGR1 | Q7Z3B1 | 1.61 | 0.000 |
| SDF4 | Q9BRK5 | 1.61 | 0.000 |
| LAMA1 | P25391 | 1.57 | 0.000 |
| BMP1 | P13497 | 1.53 | 0.000 |
| CST3 | P01034 | 1.53 | 0.003 |
| MMP14 | P50281 | 1.51 | 0.024 |
| EDIL3 | O43854 | 1.47 | 0.000 |
| GOLM1 | Q8NBJ4 | 1.43 | 0.009 |
| HTRA1 | Q92743 | 1.42 | 0.000 |
| CFH | P08603 | 1.40 | 0.000 |
| NRP1 | O14786 | 1.38 | 0.004 |
| CTHRC1 | Q96CG8 | 1.35 | 0.003 |
| GM2A | P17900 | 1.34 | 0.000 |
| SERPINE2 | P07093 | 1.33 | 0.000 |
| LUM | P51884 | 1.32 | 0.000 |
| LOX | P28300 | 1.28 | 0.000 |
| COL5A2 | P05997 | 1.27 | 0.000 |
| CCDC80 | Q76M96 | 1.27 | 0.000 |
| CDH2 | P19022 | 1.26 | 0.000 |
| POSTN | Q15063 | 1.20 | 0.000 |
| EMILIN1 | Q9Y6C2 | 1.20 | 0.000 |
| TGFBI | Q15582 | 1.19 | 0.000 |
| GALNT5 | Q7Z7M9 | 1.18 | 0.000 |
| NID2 | Q14112 | 1.16 | 0.000 |
| TIMP2 | P16035 | 1.16 | 0.002 |
| HMCN1 | Q96RW7 | 1.15 | 0.001 |
| SERPINE1 | P05121 | 1.10 | 0.000 |
| PXDN | Q92626 | 1.10 | 0.000 |
| CD109 | Q6YHK3 | 1.10 | 0.000 |
| CILP2 | Q8IUL8 | 1.00 |  |
| CLSTN1 | O94985 | 1.00 | 0.000 |
| DAG1 | Q14118 | 0.98 | 0.000 |
| SEMA7A | O75326 | 0.97 | 0.002 |
| CLU | P10909 | 0.96 | 0.003 |
| PRSS23 | O95084 | 0.94 | 0.010 |
| FSTL1 | Q12841 | 0.92 | 0.001 |
| TIMP1 | P01033 | 0.87 | 0.049 |
| CTSZ | Q9UBR2 | 0.87 | 0.006 |
| LAMB1 | P07942 | 0.83 | 0.000 |
| CCN2 | P29279 | 0.83 | 0.039 |
| HSPG2 | P98160 | 0.83 | 0.000 |
| NID1 | P14543 | 0.81 | 0.000 |
| CDH11 | P55287 | 0.80 | 0.002 |
| MFGE8 | Q08431 | 0.79 | 0.006 |
| B4GAT1 | O43505 | 0.77 | 0.015 |
| FBLN1 | P23142 | 0.75 | 0.010 |
| LAMC1 | P11047 | 0.74 | 0.000 |
| LRP1 | Q07954 | 0.71 | 0.000 |
| PSAP | P07602 | 0.70 | 0.009 |
| GAS6 | Q14393 | 0.69 | 0.019 |
| COL5A1 | P20908 | 0.65 | 0.002 |
| PTX3 | P26022 | 0.61 | 0.013 |
| NUCB1 | Q02818 | 0.60 | 0.000 |
| GGH | Q92820 | 0.57 | 0.027 |
| AEBP1 | Q8IUX7 | 0.52 | 0.002 |
| CPA4 | Q9UI42 | 0.51 | 0.016 |
| FN1 | P02751 | 0.44 | 0.000 |
| MXRA8 | Q9BRK3 | 0.41 | 0.015 |
| COL6A3 | P12111 | 0.21 | 0.001 |
| UGDH | O60701 | -0.20 |  |
| COL1A2 | P08123 | -0.39 | 0.031 |
| COL6A2 | P12110 | -0.43 | 0.014 |
| ILF3 | Q12906 | -0.54 |  |
| RCN1 | Q15293 | -0.54 | 0.041 |
| HSPE1 | P61604 | -0.60 | 0.014 |
| COL6A1 | P12109 | -0.64 | 0.000 |
| MVP | Q14764 | -0.71 |  |
| SEPTIN11 | Q9NVA2 | -0.75 | 0.036 |
| S100A9 | P06702 | -0.77 | 0.005 |
| C1S | P09871 | -0.77 | 0.007 |
| KRT76 | Q01546 | -0.79 | 0.043 |
| FKBP10 | Q96AY3 | -0.80 | 0.010 |
| TPM4 | P67936 | -0.82 | 0.012 |
| HSPD1 | P10809 | -0.83 | 0.003 |
| MAP4 | P27816 | -0.92 | 0.018 |
| AHNAK | Q09666 | -0.98 | 0.001 |
| ANPEP | P15144 | -1.01 | 0.000 |
| GAPDH | P04406 | -1.05 | 0.005 |
| WDR1 | O75083 | -1.05 | 0.028 |
| NME2 | P22392 | -1.06 | 0.002 |
| TNC | P24821 | -1.08 | 0.000 |
| MYH9 | P35579 | -1.09 | 0.002 |
| GNG12 | Q9UBI6 | -1.11 | 0.000 |
| LAMP1 | P11279 | -1.11 | 0.003 |
| AKAP12 | Q02952 | -1.12 | 0.005 |
| ANXA1 | P04083 | -1.12 | 0.000 |
| CD44 | P16070 | -1.16 | 0.012 |
| CHI3L1 | P36222 | -1.20 | 0.000 |
| BASP1 | P80723 | -1.20 | 0.000 |
| TPM3 | P06753 | -1.23 | 0.011 |
| S100A16 | Q96FQ6 | -1.34 | 0.000 |
| HSPA8 | P11142 | -1.35 | 0.000 |
| GLOD4 | Q9HC38 | -1.36 | 0.041 |
| COLEC12 | Q5KU26 | -1.37 | 0.012 |
| KRT1 | P04264 | -1.38 | 0.000 |
| DLD | P09622 | -1.38 | 0.000 |
| HSPA1B | P0DMV9 | -1.40 | 0.000 |
| PFN1 | P07737 | -1.40 | 0.000 |
| RBMX | P38159 | -1.41 | 0.000 |
| P4HB | P07237 | -1.41 | 0.000 |
| HNRNPA1L2 | Q32P51 | -1.43 | 0.001 |
| YWHAB | P31946 | -1.46 | 0.000 |
| PSME1 | Q06323 | -1.47 |  |
| HSPA6 | P17066 | -1.50 | 0.000 |
| CFL1 | P23528 | -1.50 | 0.000 |
| ALDOA | P04075 | -1.51 | 0.000 |
| IQGAP1 | P46940 | -1.53 | 0.010 |
| HSPA5 | P11021 | -1.54 | 0.000 |
| FLNB | O75369 | -1.54 | 0.002 |
| ANXA2 | P07355 | -1.55 | 0.000 |
| ENO1 | P06733 | -1.55 | 0.000 |
| UBE2NL | Q5JXB2 | -1.55 | 0.004 |
| PRDX4 | Q13162 | -1.55 | 0.000 |
| PLEC | Q15149 | -1.56 | 0.000 |
| ACTBL2 | Q562R1 | -1.58 | 0.001 |
| SFN | P31947 | -1.58 | 0.001 |
| TUBA4A | P68366 | -1.61 |  |
| DPYSL2 | Q16555 | -1.62 | 0.000 |
| CSRP1 | P21291 | -1.62 | 0.000 |
| RDX | P35241 | -1.62 | 0.000 |
| AKR1A1 | P14550 | -1.64 |  |
| TAGLN | Q01995 | -1.65 | 0.000 |
| S100A11 | P31949 | -1.67 | 0.004 |
| YWHAZ | P63104 | -1.70 | 0.000 |
| FH | P07954 | -1.72 | 0.000 |
| SH3BGRL3 | Q9H299 | -1.76 | 0.000 |
| SERPINH1 | P50454 | -1.77 | 0.001 |
| FLNC | Q14315 | -1.77 | 0.000 |
| IDE | P14735 | -1.77 |  |
| VIM | P08670 | -1.82 | 0.000 |
| MSN | P26038 | -1.82 | 0.000 |
| SELENBP1 | Q13228 | -1.82 |  |
| PRDX3 | P30048 | -1.82 | 0.000 |
| VCL | P18206 | -1.87 | 0.000 |
| GOT1 | P17174 | -1.87 | 0.000 |
| EEF1A1P5 | Q5VTE0 | -1.88 | 0.000 |
| NME1 | P15531 | -1.92 | 0.000 |
| EEF2 | P13639 | -1.95 | 0.000 |
| HNRNPK | P61978 | -2.00 |  |
| CALD1 | Q05682 | -2.00 | 0.000 |
| ATP5F1B | P06576 | -2.02 | 0.000 |
| ACTG1 | P63261 | -2.02 | 0.000 |
| COL2A1 | P02458 | -2.04 | 0.001 |
| RRBP1 | Q9P2E9 | -2.04 | 0.026 |
| HSPA4 | P34932 | -2.05 | 0.022 |
| POTEE | Q6S8J3 | -2.10 | 0.000 |
| FLNA | P21333 | -2.11 | 0.000 |
| TUBB | P07437 | -2.11 | 0.000 |
| GDI1 | P31150 | -2.11 | 0.000 |
| TKT | P29401 | -2.12 | 0.000 |
| KCTD12 | Q96CX2 | -2.13 | 0.010 |
| GOT2 | P00505 | -2.13 | 0.000 |
| LDHB | P07195 | -2.15 | 0.000 |
| CALR | P27797 | -2.17 | 0.002 |
| TUBB4B | P68371 | -2.17 | 0.000 |
| YWHAE | P62258 | -2.19 | 0.000 |
| PKM | P14618 | -2.21 | 0.000 |
| TXNRD1 | Q16881 | -2.22 | 0.000 |
| ENO2 | P09104 | -2.22 | 0.002 |
| TXNDC5 | Q8NBS9 | -2.24 | 0.000 |
| PDIA3 | P30101 | -2.25 | 0.000 |
| ARPC4 | P59998 | -2.27 | 0.000 |
| ACTN1 | P12814 | -2.30 | 0.000 |
| EZR | P15311 | -2.34 | 0.001 |
| ACTN4 | O43707 | -2.36 | 0.000 |
| YWHAG | P61981 | -2.39 | 0.001 |
| H4C1 | P62805 | -2.40 | 0.000 |
| PPIB | P23284 | -2.43 | 0.000 |
| GDI2 | P50395 | -2.44 | 0.000 |
| PDIA6 | Q15084 | -2.45 | 0.000 |
| MYL6 | P60660 | -2.46 | 0.002 |
| LDHA | P00338 | -2.50 | 0.000 |
| PRDX6 | P30041 | -2.52 |  |
| VCP | P55072 | -2.52 | 0.000 |
| GPI | P06744 | -2.53 | 0.000 |
| INA | Q16352 | -2.55 | 0.000 |
| HSPB1 | P04792 | -2.56 | 0.000 |
| LIF | P15018 | -2.60 |  |
| ANXA6 | P08133 | -2.62 | 0.004 |
| TUBB2B | Q9BVA1 | -2.69 | 0.002 |
| LGALS1 | P09382 | -2.78 | 0.000 |
| HNRNPA2B1 | P22626 | -2.79 | 0.000 |
| MDH1 | P40925 | -2.82 | 0.002 |
| GBE1 | Q04446 | -2.87 | 0.031 |
| MDH2 | P40926 | -2.89 | 0.000 |
| S100A6 | P06703 | -2.95 | 0.001 |
| CALM3 | P0DP25 | -3.02 | 0.000 |
| TPI1 | P60174 | -3.03 | 0.000 |
| COTL1 | Q14019 | -3.12 |  |
| LMNA | P02545 | -3.19 | 0.000 |
| S100A13 | Q99584 | -3.37 | 0.001 |
| TUBA1C | Q9BQE3 | -3.72 | 0.000 |
| RPLP0 | P05388 | -4.88 | 0.000 |

| **Supplementary Table 2.** MSC secretome following traumatic CM stimulation | | | |
| --- | --- | --- | --- |
| **gene_ID** | **protein_ID** | **Fold change (Log2)** | **p.value.adjusted** |
| PRG4 | Q92954 | 5.40 | 0.000 |
| ACAN | P16112 | 5.37 | 0.000 |
| COMP | P49747 | 5.26 | 0.000 |
| FNDC1 | Q4ZHG4 | 4.26 | 0.000 |
| MMP1 | P03956 | 3.30 | 0.000 |
| SERPINE2 | P07093 | 3.26 | 0.000 |
| FGG | P02679 | 2.98 |  |
| SERPINA1 | P01009 | 2.94 | 0.000 |
| TGFBI | Q15582 | 2.80 | 0.000 |
| ALB | P02768 | 2.79 | 0.000 |
| CHAD | O15335 | 2.72 |  |
| TIMP1 | P01033 | 2.54 | 0.000 |
| FGB | P02675 | 2.50 |  |
| CLU | P10909 | 2.42 | 0.000 |
| CTHRC1 | Q96CG8 | 2.41 | 0.000 |
| FAM3C | Q92520 | 2.34 | 0.000 |
| CILP | O75339 | 2.33 |  |
| SEMA7A | O75326 | 2.28 | 0.000 |
| LUM | P51884 | 2.18 | 0.000 |
| SRGN | P10124 | 2.16 | 0.001 |
| CXCL12 | P48061 | 2.10 | 0.001 |
| HTRA1 | Q92743 | 2.05 | 0.000 |
| FBN1 | P35555 | 2.04 | 0.000 |
| CTSB | P07858 | 2.04 | 0.000 |
| FGA | P02671 | 2.03 |  |
| PXDN | Q92626 | 2.02 | 0.000 |
| SRPX | P78539 | 1.89 | 0.000 |
| SPOCK1 | Q08629 | 1.89 | 0.000 |
| GALNT1 | Q10472 | 1.83 | 0.000 |
| STC2 | O76061 | 1.83 | 0.000 |
| CCDC80 | Q76M96 | 1.80 | 0.000 |
| LTBP1 | Q14766 | 1.78 | 0.000 |
| CDH2 | P19022 | 1.74 | 0.000 |
| LTBP2 | Q14767 | 1.70 | 0.000 |
| VCAN | P13611 | 1.69 | 0.000 |
| MAN1A1 | P33908 | 1.65 | 0.000 |
| COL7A1 | Q02388 | 1.63 | 0.000 |
| SRPX2 | O60687 | 1.59 | 0.000 |
| SBSN | Q6UWP8 | 1.54 | 0.027 |
| DCN | P07585 | 1.52 | 0.000 |
| LAMA1 | P25391 | 1.50 | 0.000 |
| B2M | P61769 | 1.50 | 0.003 |
| GALNT5 | Q7Z7M9 | 1.48 | 0.000 |
| ABI3BP | Q7Z7G0 | 1.48 | 0.000 |
| TNC | P24821 | 1.48 | 0.000 |
| SEMG2 | Q02383 | 1.43 |  |
| APP | P05067 | 1.39 | 0.000 |
| OGN | P20774 | 1.39 |  |
| VASN | Q6EMK4 | 1.37 | 0.000 |
| NUCB2 | P80303 | 1.37 | 0.000 |
| SMOC1 | Q9H4F8 | 1.36 |  |
| APOE | P02649 | 1.34 | 0.000 |
| CLSTN1 | O94985 | 1.33 | 0.000 |
| CFH | P08603 | 1.30 | 0.000 |
| CLEC11A | Q9Y240 | 1.29 | 0.003 |
| GOLM1 | Q8NBJ4 | 1.29 | 0.000 |
| AEBP1 | Q8IUX7 | 1.28 | 0.000 |
| ITIH1 | P19827 | 1.23 |  |
| ANGPT1 | Q15389 | 1.22 | 0.046 |
| BMP1 | P13497 | 1.21 | 0.000 |
| HMCN1 | Q96RW7 | 1.17 | 0.000 |
| CCN2 | P29279 | 1.16 | 0.000 |
| IGFBP7 | Q16270 | 1.16 | 0.009 |
| TPP1 | O14773 | 1.13 | 0.000 |
| MANBA | O00462 | 1.12 | 0.015 |
| COL4A2 | P08572 | 1.12 | 0.001 |
| GAS6 | Q14393 | 1.11 | 0.000 |
| EFEMP1 | Q12805 | 1.11 | 0.007 |
| THBS1 | P07996 | 1.11 | 0.000 |
| IGHA1 | P01876 | 1.10 |  |
| MMP2 | P08253 | 1.07 | 0.000 |
| QSOX1 | O00391 | 1.07 | 0.000 |
| PRSS23 | O95084 | 1.04 | 0.045 |
| PLG | P00747 | 1.03 |  |
| ADAM9 | Q13443 | 1.01 | 0.012 |
| EMILIN1 | Q9Y6C2 | 1.01 | 0.000 |
| SERPINE1 | P05121 | 1.00 | 0.000 |
| POSTN | Q15063 | 0.99 | 0.000 |
| CTSL | P07711 | 0.99 | 0.015 |
| NID2 | Q14112 | 0.97 | 0.000 |
| FBLN1 | P23142 | 0.96 | 0.005 |
| EDIL3 | O43854 | 0.95 | 0.000 |
| PTK7 | Q13308 | 0.95 | 0.001 |
| THBS2 | P35442 | 0.93 | 0.000 |
| LAMB1 | P07942 | 0.93 | 0.000 |
| COL11A1 | P12107 | 0.92 | 0.000 |
| PROS1 | P07225 | 0.91 | 0.004 |
| COL5A2 | P05997 | 0.87 | 0.008 |
| IL6 | P05231 | 0.87 |  |
| CTSD | P07339 | 0.86 | 0.000 |
| EFEMP2 | O95967 | 0.85 | 0.017 |
| COL5A1 | P20908 | 0.83 | 0.000 |
| LAMC1 | P11047 | 0.83 | 0.000 |
| SDF4 | Q9BRK5 | 0.81 | 0.032 |
| OLFML3 | Q9NRN5 | 0.79 | 0.000 |
| ECM1 | Q16610 | 0.77 | 0.000 |
| NUCB1 | Q02818 | 0.75 | 0.000 |
| BGN | P21810 | 0.73 | 0.001 |
| MXRA8 | Q9BRK3 | 0.71 | 0.010 |
| HPX | P02790 | 0.70 |  |
| NRP1 | O14786 | 0.68 | 0.000 |
| CEMIP | Q8WUJ3 | 0.68 | 0.003 |
| NID1 | P14543 | 0.64 | 0.004 |
| PLTP | P55058 | 0.63 | 0.048 |
| LOXL2 | Q9Y4K0 | 0.63 | 0.002 |
| CTSA | P10619 | 0.63 | 0.030 |
| CFB | P00751 | 0.58 | 0.007 |
| MMP13 | P45452 | 0.53 |  |
| GGH | Q92820 | 0.53 | 0.007 |
| HSPG2 | P98160 | 0.49 | 0.000 |
| CRLF1 | O75462 | 0.48 |  |
| LAMA4 | Q16363 | 0.47 | 0.001 |
| COL6A1 | P12109 | 0.42 | 0.003 |
| C1R | P00736 | 0.38 | 0.021 |
| FN1 | P02751 | 0.37 | 0.000 |
| COL6A2 | P12110 | 0.35 | 0.009 |
| ANXA1 | P04083 | 0.34 | 0.014 |
| CD109 | Q6YHK3 | 0.30 | 0.012 |
| MMP10 | P09238 | 0.26 |  |
| ENO3 | P13929 | 0.26 |  |
| LGALS7; LGALS7B | P47929 | 0.26 |  |
| CHI3L2 | Q15782 | 0.26 |  |
| LBP | P18428 | 0.05 |  |
| YWHAQ | P27348 | 0.03 |  |
| COL6A3 | P12111 | -0.15 | 0.007 |
| PRDX1 | Q06830 | -0.32 | 0.003 |
| TGFB2 | P61812 | -0.34 |  |
| P4HB | P07237 | -0.36 | 0.035 |
| PKM | P14618 | -0.38 | 0.009 |
| COTL1 | Q14019 | -0.39 | 0.043 |
| PDIA3 | P30101 | -0.41 | 0.001 |
| S100A11 | P31949 | -0.42 | 0.050 |
| HSP90B1 | P14625 | -0.47 | 0.001 |
| GANAB | Q14697 | -0.48 | 0.004 |
| SERPING1 | P05155 | -0.48 | 0.005 |
| VCL | P18206 | -0.50 | 0.001 |
| PRDX2 | P32119 | -0.51 | 0.033 |
| PLEC | Q15149 | -0.52 | 0.000 |
| PDIA4 | P13667 | -0.55 | 0.001 |
| ACTN4 | O43707 | -0.56 | 0.006 |
| RCN1 | Q15293 | -0.56 | 0.004 |
| PDIA6 | Q15084 | -0.58 | 0.015 |
| CALD1 | Q05682 | -0.58 | 0.028 |
| LASP1 | Q14847 | -0.58 | 0.001 |
| SPON2 | Q9BUD6 | -0.60 | 0.035 |
| H4C1 | P62805 | -0.60 | 0.046 |
| HSP90AA1 | P07900 | -0.64 | 0.027 |
| PTGDS | P41222 | -0.67 | 0.012 |
| PGK1 | P00558 | -0.68 | 0.010 |
| HSPB1 | P04792 | -0.69 | 0.049 |
| ENO1 | P06733 | -0.69 | 0.000 |
| TPM4 | P67936 | -0.75 | 0.000 |
| YWHAZ | P63104 | -0.76 | 0.000 |
| MYH9 | P35579 | -0.76 | 0.000 |
| PFN1 | P07737 | -0.76 | 0.002 |
| HSPE1 | P61604 | -0.78 | 0.006 |
| PRSS1 | P07477 | -0.81 | 0.002 |
| TAGLN | Q01995 | -0.83 | 0.001 |
| GPI | P06744 | -0.84 | 0.000 |
| LMNA | P02545 | -0.92 | 0.000 |
| HSPD1 | P10809 | -0.92 | 0.010 |
| COL14A1 | Q05707 | -0.99 | 0.000 |
| DCD | P81605 | -0.99 | 0.001 |
| FABP1 | P07148 | -1.01 | 0.021 |
| MDH1 | P40925 | -1.04 | 0.000 |
| S100A6 | P06703 | -1.14 | 0.011 |
| ALDH1A1 | P00352 | -1.23 | 0.011 |
| CALR | P27797 | -1.28 | 0.000 |
| TKT | P29401 | -1.29 | 0.000 |
| GLUD2 | P49448 | -1.31 | 0.000 |
| TPI1 | P60174 | -1.33 | 0.000 |
| ATP5F1A | P25705 | -1.40 | 0.000 |
| TUBA1C | Q9BQE3 | -1.57 | 0.000 |
| MDH2 | P40926 | -1.59 | 0.000 |
| TUBB | P07437 | -1.77 | 0.000 |
| ACTA1 | P68133 | -1.85 | 0.032 |
| COL2A1 | P02458 | -2.20 | 0.004 |
| APOA1 | P02647 | -2.80 | 0.013 |
| CLIC1 | O00299 | -3.06 |  |

| **Supplementary Table 3.** MSC secretome following degenerative CM stimulation | | | |
| --- | --- | --- | --- |
| **gene_ID** | **protein_ID** | **Fold change (Log2)** | **p.value.adjusted** |
| PRG4 | Q92954 | 7.36 | 0.000 |
| ACAN | P16112 | 5.78 | 0.000 |
| COMP | P49747 | 5.36 | 0.000 |
| FNDC1 | Q4ZHG4 | 4.69 | 0.000 |
| SERPINA1 | P01009 | 3.63 | 0.000 |
| SRGN | P10124 | 3.16 | 0.000 |
| MMP1 | P03956 | 3.13 | 0.000 |
| FGG | P02679 | 2.85 |  |
| CHAD | O15335 | 2.83 |  |
| SERPINE2 | P07093 | 2.76 | 0.000 |
| SERPINE1 | P05121 | 2.74 | 0.000 |
| CTHRC1 | Q96CG8 | 2.67 | 0.000 |
| DCN | P07585 | 2.66 | 0.000 |
| CTSB | P07858 | 2.65 | 0.000 |
| TIMP1 | P01033 | 2.57 | 0.000 |
| CLU | P10909 | 2.57 | 0.000 |
| LUM | P51884 | 2.56 | 0.000 |
| TGFBI | Q15582 | 2.52 | 0.000 |
| FGB | P02675 | 2.44 |  |
| ALB | P02768 | 2.33 | 0.000 |
| STC2 | O76061 | 2.31 | 0.000 |
| FGA | P02671 | 2.28 |  |
| ABI3BP | Q7Z7G0 | 2.22 | 0.000 |
| LAMA1 | P25391 | 2.16 | 0.000 |
| KRT77 | Q7Z794 | 2.15 | 0.048 |
| CILP | O75339 | 2.07 |  |
| FAM3C | Q92520 | 2.06 | 0.000 |
| SEMA7A | O75326 | 2.05 | 0.000 |
| HPX | P02790 | 2.05 |  |
| SBSN | Q6UWP8 | 2.04 | 0.003 |
| KRT78 | Q8N1N4 | 2.02 | 0.043 |
| SRPX2 | O60687 | 2.00 | 0.000 |
| ITIH1 | P19827 | 1.96 |  |
| LGALS7 | P47929 | 1.94 |  |
| MAN1A1 | P33908 | 1.90 | 0.000 |
| IGHA1 | P01876 | 1.88 |  |
| B2M | P61769 | 1.86 | 0.000 |
| HTRA1 | Q92743 | 1.85 | 0.000 |
| PXDN | Q92626 | 1.85 | 0.000 |
| MMP3 | P08254 | 1.84 | 0.000 |
| CLSTN1 | O94985 | 1.83 | 0.000 |
| LTBP1 | Q14766 | 1.76 | 0.000 |
| VCAN | P13611 | 1.75 | 0.000 |
| COL7A1 | Q02388 | 1.74 | 0.000 |
| SRPX | P78539 | 1.69 | 0.000 |
| CDH2 | P19022 | 1.68 | 0.000 |
| SMOC1 | Q9H4F8 | 1.64 |  |
| ENO3 | P13929 | 1.63 |  |
| VASN | Q6EMK4 | 1.62 | 0.000 |
| SPOCK1 | Q08629 | 1.62 | 0.000 |
| KRT16 | P08779 | 1.59 | 0.008 |
| CXCL12 | P48061 | 1.55 | 0.010 |
| BMP1 | P13497 | 1.48 | 0.000 |
| LTBP2 | Q14767 | 1.47 | 0.000 |
| CCDC80 | Q76M96 | 1.46 | 0.000 |
| APOE | P02649 | 1.42 | 0.000 |
| FBN1 | P35555 | 1.41 | 0.000 |
| GALNT1 | Q10472 | 1.39 | 0.000 |
| OGN | P20774 | 1.34 |  |
| CCN1 | O00622 | 1.32 | 0.001 |
| TTN | Q8WZ42 | 1.32 | 0.007 |
| DSP | P15924 | 1.29 | 0.000 |
| MMP13 | P45452 | 1.27 |  |
| GAS6 | Q14393 | 1.27 | 0.000 |
| PTK7 | Q13308 | 1.26 | 0.000 |
| KRT17 | Q04695 | 1.26 | 0.033 |
| CFB | P00751 | 1.25 | 0.000 |
| GOLM1 | Q8NBJ4 | 1.24 | 0.000 |
| IL6 | P05231 | 1.22 |  |
| QSOX1 | O00391 | 1.22 | 0.000 |
| COL4A2 | P08572 | 1.20 | 0.000 |
| CFH | P08603 | 1.15 | 0.000 |
| PLG | P00747 | 1.15 |  |
| APP | P05067 | 1.14 | 0.000 |
| MANBA | O00462 | 1.14 | 0.015 |
| GALNT5 | Q7Z7M9 | 1.11 | 0.000 |
| PRSS23 | O95084 | 1.10 | 0.021 |
| THBS1 | P07996 | 1.10 | 0.000 |
| EFEMP1 | Q12805 | 1.09 | 0.005 |
| COL11A1 | P12107 | 1.09 | 0.000 |
| MMP2 | P08253 | 1.08 | 0.000 |
| AEBP1 | Q8IUX7 | 1.05 | 0.000 |
| BGN | P21810 | 1.03 | 0.000 |
| ADAM9 | Q13443 | 1.00 | 0.009 |
| IGFBP7 | Q16270 | 1.00 | 0.021 |
| NUCB2 | P80303 | 0.99 | 0.000 |
| CCN2 | P29279 | 0.97 | 0.000 |
| KRT5 | P13647 | 0.94 | 0.046 |
| COL5A1 | P20908 | 0.94 | 0.000 |
| TPP1 | O14773 | 0.92 | 0.001 |
| MXRA8 | Q9BRK3 | 0.91 | 0.001 |
| FSTL1 | Q12841 | 0.87 | 0.002 |
| POSTN | Q15063 | 0.85 | 0.000 |
| CTSL | P07711 | 0.85 | 0.043 |
| FBLN1 | P23142 | 0.83 | 0.015 |
| PROS1 | P07225 | 0.80 | 0.007 |
| HSPG2 | P98160 | 0.79 | 0.000 |
| EDIL3 | O43854 | 0.79 | 0.000 |
| FN1 | P02751 | 0.78 | 0.000 |
| KRT1 | P04264 | 0.77 | 0.002 |
| CEMIP | Q8WUJ3 | 0.76 | 0.001 |
| MMP13 | P45452 | 0.74 |  |
| CHI3L1 | P36222 | 0.73 | 0.001 |
| NID2 | Q14112 | 0.71 | 0.000 |
| TNC | P24821 | 0.71 | 0.000 |
| NUCB1 | Q02818 | 0.69 | 0.000 |
| OLFML3 | Q9NRN5 | 0.69 | 0.000 |
| EFEMP2 | O95967 | 0.67 | 0.046 |
| C1R | P00736 | 0.65 | 0.000 |
| EMILIN1 | Q9Y6C2 | 0.65 | 0.001 |
| GGH | Q92820 | 0.64 | 0.001 |
| KRT9 | P35527 | 0.63 | 0.001 |
| VCAM1 | P19320 | 0.62 | 0.000 |
| KRT2 | P35908 | 0.61 | 0.004 |
| THBS2 | P35442 | 0.61 | 0.000 |
| LOXL2 | Q9Y4K0 | 0.57 | 0.003 |
| NID1 | P14543 | 0.56 | 0.005 |
| CTSD | P07339 | 0.56 | 0.002 |
| CDH11 | P55287 | 0.55 | 0.006 |
| C1S | P09871 | 0.51 | 0.006 |
| LAMC1 | P11047 | 0.50 | 0.000 |
| ALDH1A1 | P00352 | 0.49 | 0.048 |
| LAMB1 | P07942 | 0.49 | 0.000 |
| ECM1 | Q16610 | 0.46 | 0.002 |
| LBP | P18428 | 0.40 |  |
| CD109 | Q6YHK3 | 0.33 | 0.005 |
| COL6A1 | P12109 | 0.27 | 0.038 |
| TGFB2 | P61812 | 0.26 |  |
| COL6A2 | P12110 | 0.26 | 0.046 |
| SEMG2 | Q02383 | 0.22 |  |
| CHI3L2 | Q15782 | 0.22 |  |
| CRLF1 | O75462 | 0.01 |  |
| COL6A3 | P12111 | -0.26 | 0.000 |
| ANXA2 | P07355 | -0.29 | 0.008 |
| ALDOA | P04075 | -0.34 | 0.008 |
| UBA52 | P62987 | -0.36 | 0.015 |
| YWHAQ | P27348 | -0.37 |  |
| MSN | P26038 | -0.38 | 0.004 |
| GDI2 | P50395 | -0.40 | 0.009 |
| CD248 | Q9HCU0 | -0.43 | 0.005 |
| TPM3 | P06753 | -0.43 | 0.016 |
| ANPEP | P15144 | -0.48 | 0.000 |
| SERPING1 | P05155 | -0.48 | 0.004 |
| LDHA | P00338 | -0.48 | 0.016 |
| RCN1 | Q15293 | -0.51 | 0.002 |
| PPIB | P23284 | -0.52 | 0.021 |
| TXNDC5 | Q8NBS9 | -0.54 | 0.000 |
| PRDX2 | P32119 | -0.55 | 0.014 |
| HSPA5 | P11021 | -0.56 | 0.000 |
| YWHAE | P62258 | -0.58 | 0.007 |
| LDHB | P07195 | -0.58 | 0.024 |
| FLNA | P21333 | -0.60 | 0.000 |
| AHNAK | Q09666 | -0.61 | 0.048 |
| COTL1 | Q14019 | -0.62 | 0.002 |
| PDIA3 | P30101 | -0.62 | 0.000 |
| PRDX1 | Q06830 | -0.63 | 0.000 |
| PRSS1 | P07477 | -0.63 | 0.009 |
| PFN1 | P07737 | -0.64 | 0.008 |
| LASP1 | Q14847 | -0.66 | 0.000 |
| LAMB2 | P55268 | -0.66 | 0.000 |
| CALD1 | Q05682 | -0.69 | 0.003 |
| P4HB | P07237 | -0.70 | 0.000 |
| HSPE1 | P61604 | -0.71 | 0.021 |
| MDH1 | P40925 | -0.73 | 0.000 |
| SFN | P31947 | -0.76 | 0.004 |
| VCL | P18206 | -0.77 | 0.000 |
| PSAP | P07602 | -0.78 | 0.041 |
| ACTN4 | O43707 | -0.83 | 0.000 |
| FSCN1 | Q16658 | -0.85 | 0.007 |
| GNB4 | Q9HAV0 | -0.85 | 0.002 |
| YWHAZ | P63104 | -0.86 | 0.000 |
| ANXA5 | P08758 | -0.89 | 0.000 |
| GPI | P06744 | -0.89 | 0.000 |
| RRBP1 | Q9P2E9 | -0.90 | 0.001 |
| EEF2 | P13639 | -0.92 | 0.013 |
| TAGLN2 | P37802 | -0.92 | 0.001 |
| ATP5F1A | P25705 | -0.95 | 0.014 |
| GLUD2 | P49448 | -0.96 | 0.004 |
| SERPINH1 | P50454 | -0.96 | 0.014 |
| ENO1 | P06733 | -0.99 | 0.000 |
| S100A11 | P31949 | -0.99 | 0.000 |
| PDIA4 | P13667 | -1.11 | 0.000 |
| RPL7A | P62424 | -1.11 | 0.003 |
| TPM4 | P67936 | -1.12 | 0.000 |
| GANAB | Q14697 | -1.13 | 0.000 |
| PLEC | Q15149 | -1.13 | 0.000 |
| GAPDH | P04406 | -1.13 | 0.011 |
| SPON2 | Q9BUD6 | -1.13 | 0.001 |
| TKT | P29401 | -1.14 | 0.000 |
| ACTG1 | P63261 | -1.16 | 0.000 |
| HSP90B1 | P14625 | -1.16 | 0.000 |
| CALR | P27797 | -1.16 | 0.000 |
| LGALS1 | P09382 | -1.19 | 0.000 |
| PKM | P14618 | -1.20 | 0.000 |
| MYL6 | P60660 | -1.20 | 0.000 |
| TAGLN | Q01995 | -1.21 | 0.000 |
| TPI1 | P60174 | -1.25 | 0.000 |
| VIM | P08670 | -1.27 | 0.000 |
| HSP90AA1 | P07900 | -1.27 | 0.000 |
| PGK1 | P00558 | -1.28 | 0.000 |
| CFL1 | P23528 | -1.28 | 0.000 |
| EEF1A1P5 | Q5VTE0 | -1.30 | 0.000 |
| MYO7B | Q6PIF6 | -1.32 | 0.020 |
| HSPD1 | P10809 | -1.32 | 0.002 |
| HSP90AB1 | P08238 | -1.45 | 0.000 |
| PDIA6 | Q15084 | -1.50 | 0.000 |
| MYH9 | P35579 | -1.50 | 0.000 |
| FASN | P49327 | -1.52 | 0.003 |
| RPLP0 | P05388 | -1.55 | 0.000 |
| H4C1 | P62805 | -1.55 | 0.000 |
| S100A6 | P06703 | -1.56 | 0.000 |
| H2BC13 | Q99880 | -1.57 | 0.000 |
| LMNA | P02545 | -1.59 | 0.000 |
| COL14A1 | Q05707 | -1.64 | 0.000 |
| TUBB | P07437 | -1.82 | 0.000 |
| MDH2 | P40926 | -1.82 | 0.000 |
| COL2A1 | P02458 | -1.95 | 0.010 |
| TUBA1C | Q9BQE3 | -2.09 | 0.000 |
| NCL | P19338 | -2.22 | 0.000 |
| RPSA | P08865 | -2.23 | 0.018 |
| CKM | P06732 | -2.35 | 0.018 |
| CLIC1 | O00299 | -3.34 |  |
| S100A8 | P05109 | -3.34 | 0.019 |

| **Supplementary Table 4.** MSC secretome following IL-1β stimulation | | | |
| --- | --- | --- | --- |
| **gene_ID** | **protein_ID** | **Fold change (Log2)** | **p.value.adjusted** |
| MMP3 | P08254 | 5.46 | 0.000 |
| CFB | P00751 | 4.75 | 0.000 |
| MMP1 | P03956 | 4.35 | 0.000 |
| IL6 | P05231 | 3.58 |  |
| SERPINA1 | P01009 | 3.01 | 0.000 |
| C3 | P01024 | 2.81 | 0.000 |
| FNDC1 | Q4ZHG4 | 2.77 | 0.000 |
| MMP10 | P09238 | 2.53 |  |
| KRT77 | Q7Z794 | 2.53 | 0.023 |
| MAN1A1 | P33908 | 2.45 | 0.000 |
| KRT78 | Q8N1N4 | 2.45 | 0.022 |
| ABI3BP | Q7Z7G0 | 2.22 | 0.000 |
| MYH7 | P12883 | 2.22 | 0.001 |
| CXCL12 | P48061 | 2.04 | 0.006 |
| SRGN | P10124 | 2.04 | 0.003 |
| B2M | P61769 | 2.02 | 0.000 |
| SERPINE1 | P05121 | 1.90 | 0.000 |
| STC2 | O76061 | 1.88 | 0.000 |
| ALDH1A1 | P00352 | 1.85 | 0.007 |
| LBP | P18428 | 1.82 |  |
| FABP1 | P07148 | 1.79 | 0.004 |
| CHI3L1 | P36222 | 1.77 | 0.000 |
| IGHA1 | P01876 | 1.71 |  |
| VCAM1 | P19320 | 1.65 | 0.000 |
| CHI3L2 | Q15782 | 1.58 |  |
| TNC | P24821 | 1.55 | 0.000 |
| SERPINE2 | P07093 | 1.48 | 0.000 |
| ENO3 | P13929 | 1.47 |  |
| LUM | P51884 | 1.36 | 0.000 |
| C1R | P00736 | 1.32 | 0.000 |
| CFH | P08603 | 1.20 | 0.000 |
| SRPX2 | O60687 | 1.20 | 0.003 |
| COL7A1 | Q02388 | 1.18 | 0.000 |
| MANBA | O00462 | 1.18 | 0.022 |
| C1S | P09871 | 1.15 | 0.000 |
| CLSTN1 | O94985 | 1.13 | 0.000 |
| CDH2 | P19022 | 1.13 | 0.000 |
| NID2 | Q14112 | 1.09 | 0.000 |
| NUCB2 | P80303 | 1.08 | 0.000 |
| QSOX1 | O00391 | 1.01 | 0.000 |
| BMP1 | P13497 | 1.00 | 0.000 |
| VASN | Q6EMK4 | 0.97 | 0.000 |
| CTSB | P07858 | 0.89 | 0.022 |
| LAMA1 | P25391 | 0.89 | 0.001 |
| FSTL1 | Q12841 | 0.87 | 0.003 |
| SDF4 | Q9BRK5 | 0.84 | 0.032 |
| PTX3 | P26022 | 0.83 | 0.004 |
| GOLM1 | Q8NBJ4 | 0.83 | 0.000 |
| SRPX | P78539 | 0.82 | 0.012 |
| FBLN1 | P23142 | 0.80 | 0.020 |
| HEXA | P06865 | 0.78 | 0.034 |
| CTHRC1 | Q96CG8 | 0.77 | 0.000 |
| TIMP1 | P01033 | 0.76 | 0.004 |
| TGFBI | Q15582 | 0.69 | 0.000 |
| LGALS3BP | Q08380 | 0.66 | 0.000 |
| GALNT5 | Q7Z7M9 | 0.65 | 0.000 |
| CTSD | P07339 | 0.63 | 0.001 |
| POSTN | Q15063 | 0.60 | 0.002 |
| TPP1 | O14773 | 0.59 | 0.043 |
| APP | P05067 | 0.58 | 0.002 |
| CALU | O43852 | 0.58 | 0.002 |
| FBN1 | P35555 | 0.56 | 0.004 |
| COL3A1 | P02461 | 0.56 | 0.005 |
| KRT1 | P04264 | 0.55 | 0.039 |
| CCDC80 | Q76M96 | 0.55 | 0.004 |
| GAS6 | Q14393 | 0.54 | 0.002 |
| COL5A1 | P20908 | 0.53 | 0.000 |
| MMP2 | P08253 | 0.51 | 0.000 |
| APOE | P02649 | 0.50 | 0.006 |
| PXDN | Q92626 | 0.47 | 0.022 |
| EDIL3 | O43854 | 0.46 | 0.000 |
| LOXL2 | Q9Y4K0 | 0.46 | 0.026 |
| GALNT1 | Q10472 | 0.41 | 0.019 |
| HSPG2 | P98160 | 0.41 | 0.000 |
| CD109 | Q6YHK3 | 0.40 | 0.001 |
| NUCB1 | Q02818 | 0.39 | 0.003 |
| FN1 | P02751 | 0.31 | 0.000 |
| COL12A1 | Q99715 | 0.20 | 0.001 |
| CHAD | O15335 | 0.07 |  |
| CILP | O75339 | 0.07 |  |
| CRLF1 | O75462 | 0.07 |  |
| PLG | P00747 | 0.07 |  |
| FGA | P02671 | 0.07 |  |
| FGB | P02675 | 0.07 |  |
| FGG | P02679 | 0.07 |  |
| ITIH1 | P19827 | 0.07 |  |
| OGN | P20774 | 0.07 |  |
| LGALS7 | P47929 | 0.07 |  |
| SEMG2 | Q02383 | 0.07 |  |
| SMOC1 | Q9H4F8 | 0.07 |  |
| MMP13 | P45452 | -0.05 |  |
| COL6A3 | P12111 | -0.23 | 0.000 |
| HPX | P02790 | -0.28 |  |
| CD248 | Q9HCU0 | -0.33 | 0.023 |
| TXNDC5 | Q8NBS9 | -0.33 | 0.024 |
| COL14A1 | Q05707 | -0.36 | 0.000 |
| LASP1 | Q14847 | -0.41 | 0.010 |
| GDI2 | P50395 | -0.43 | 0.009 |
| PDIA3 | P30101 | -0.43 | 0.001 |
| P4HB | P07237 | -0.44 | 0.016 |
| PRDX1 | Q06830 | -0.46 | 0.000 |
| VCL | P18206 | -0.47 | 0.002 |
| TIMP2 | P16035 | -0.48 | 0.026 |
| UBA52 | P62987 | -0.49 | 0.002 |
| ANPEP | P15144 | -0.49 | 0.000 |
| HSPA5 | P11021 | -0.51 | 0.000 |
| TGFB2 | P61812 | -0.51 |  |
| LRP1 | Q07954 | -0.51 | 0.000 |
| YWHAQ | P27348 | -0.55 |  |
| PRSS1 | P07477 | -0.55 | 0.032 |
| GPI | P06744 | -0.56 | 0.007 |
| TAGLN2 | P37802 | -0.58 | 0.048 |
| PDIA4 | P13667 | -0.59 | 0.000 |
| SFN | P31947 | -0.63 | 0.020 |
| FLNA | P21333 | -0.64 | 0.000 |
| YWHAE | P62258 | -0.66 | 0.004 |
| GSN | P06396 | -0.66 | 0.003 |
| S100A11 | P31949 | -0.69 | 0.002 |
| TPM3 | P06753 | -0.70 | 0.000 |
| AHNAK | Q09666 | -0.74 | 0.021 |
| RRBP1 | Q9P2E9 | -0.74 | 0.005 |
| TKT | P29401 | -0.75 | 0.000 |
| PFN1 | P07737 | -0.76 | 0.002 |
| ANXA1 | P04083 | -0.76 | 0.000 |
| TAGLN | Q01995 | -0.78 | 0.001 |
| TPM4 | P67936 | -0.79 | 0.000 |
| ENO1 | P06733 | -0.85 | 0.000 |
| CALR | P27797 | -0.89 | 0.000 |
| ANXA2 | P07355 | -0.89 | 0.000 |
| HSP90B1 | P14625 | -0.91 | 0.000 |
| MYL6 | P60660 | -0.94 | 0.002 |
| PLEC | Q15149 | -0.95 | 0.000 |
| GNB4 | Q9HAV0 | -0.96 | 0.010 |
| ATP5F1A | P25705 | -0.97 | 0.005 |
| RPL7A | P62424 | -0.99 | 0.015 |
| YWHAZ | P63104 | -0.99 | 0.000 |
| HSP90AB1 | P08238 | -1.03 | 0.032 |
| GANAB | Q14697 | -1.06 | 0.000 |
| LMNA | P02545 | -1.07 | 0.000 |
| MYH9 | P35579 | -1.13 | 0.000 |
| PDIA6 | Q15084 | -1.14 | 0.030 |
| VIM | P08670 | -1.17 | 0.000 |
| ANXA5 | P08758 | -1.23 | 0.000 |
| HSPD1 | P10809 | -1.25 | 0.002 |
| ACTG1 | P63261 | -1.31 | 0.000 |
| HSPE1 | P61604 | -1.32 | 0.001 |
| PKM | P14618 | -1.37 | 0.000 |
| MDH2 | P40926 | -1.39 | 0.002 |
| H4C1 | P62805 | -1.40 | 0.000 |
| CFL1 | P23528 | -1.43 | 0.000 |
| TUBB | P07437 | -1.44 | 0.002 |
| EEF1A1P5 | Q5VTE0 | -1.49 | 0.000 |
| HSP90AA1 | P07900 | -1.50 | 0.000 |
| GAPDH | P04406 | -1.52 | 0.001 |
| PGK1 | P00558 | -1.57 | 0.000 |
| LGALS1 | P09382 | -1.60 | 0.000 |
| EEF2 | P13639 | -1.61 | 0.000 |
| TUBA1C | Q9BQE3 | -1.68 | 0.000 |
| NCL | P19338 | -2.51 | 0.001 |
| APOA1 | P02647 | -2.78 | 0.031 |
