# Supplementary material for: Uncovering the secretome of mesenchymal stromal cells exposed to healthy, traumatic, and degenerative intervertebral discs: a proteomic analysis
Source: Stem Cell Res Ther. 2021 Jan 7;12:11. doi: 10.1186/s13287-020-02062-2 (PMC7789679; doi:10.1186/s13287-020-02062-2)
Supplement: Supplementary file 4 — Additional file 4 : Supplementary Table 5 Concentrations of cytokines and chemokines in pooled conditioned media from healthy, traumatic and degenerative intervertebral disc, measured by immunoassay technique (mean+/-sd of technical replicates; pg/mL). [file 13287_2020_2062_MOESM4_ESM.docx]

**Supplementary Table 5.** Concentrations of cytokines and chemokines in pooled conditioned media from healthy, traumatic and degenerative intervertebral disc, measured by immunoassay technique (mean+/-sd of technical replicates; pg/mL).

|  | **healthy** | **traumatic** | **degenerative** |
| --- | --- | --- | --- |
| VEGF | 3619.59±5.70 | 3326.55±58.11 | 3412.64±66.70 |
| TNFb | 1.48±0.47 | 1.16±0.09 | 0.70±0.21 |
| TNFa | 1.61±0.83 | 6.01±0.35 | 7.14±0.63 |
| TARC | 103.88±23.32 | 48.49±14.27 | 41.65±9.75 |
| MCP-4 | 137.62±21.43 | 55.57±12.27 | 31.05±7.17 |
| MCP-1 | 577.67±77.30 | 554.54±27.33 | 468.21±34.47 |
| MIP-1b | 15.56±0.67 | 23.19±4.36 | 116.50±7.43 |
| MIP-1a | 22.95±0.91 | 49.48±0.39 | 80.59±5.96 |
| MDC | 493.77±42.92 | 168.11±42.44 | 225.64±67.35 |
| IP-10 | 1570.83±164.42 | 793.13±40.75 | 691.24±36.66 |
| IL-8 | 199.49±33.75 | 2521.33±505.85 | 2492.55±8.30 |
| IL-7 | 1.97±0.37 | 0.84±0.14 | 1.46±0.17 |
| IL-6 | 399.59±12.29 | 1258.23±5.96 | 902.67±129.92 |
| IL-5 | 0.46±0.07 | 0.17±0.06 | 0.45±0.33 |
| IL-4 | 0.26±0.008 | 1.01±0.23 | 0.57±0.21 |
| IL-2 | 1.09±0.04 | 5.80±1.04 | 3.73±0.94 |
| IL-1α | 1.48±0.55 | 5.42±0.35 | 6.52±0.13 |
| IL-1b | 1.01±0.16 | 4.11±0.30 | 5.12±1.18 |
| IL-17A | 0.40±0.04 | 0.11±0.006 | 0.10±0.007 |
| IL-16 | 17.52±0.14 | 59.48±17.90 | 18.37±3.39 |
| IL-15 | 2.71±0.11 | 0.92±0.53 | 1.09±0.31 |
| IL-13 | 7.11±0.31 | 11.70±0.57 | 16.18±3.17 |
| IL-12p70 | 1.02±0.27 | 2.88±1.04 | 1.13±0.26 |
| IL-12 IL-23p40 | 1.31±0.10 | 1.54±0.40 | 0.64±0.19 |
| IL-10 | 0.48±0.008 | 325.76±158.38 | 499.06±91.08 |
| IFN-γ | 2.70±0.32 | 10.66±0.36 | 8.83±0.73 |
| GM-CSF | 0.70±0.07 | 3.26±0.85 | 9.76±3.00 |
| Eotaxin-3 | 26.86±2.36 | 14.55±2.54 | 12.70±2.33 |
| Eotaxin | 660.82±18.99 | 179.16±11.43 | 208.41±4.01 |
